# Supplementary material for: Feasibility and impact of haplogroup matching for mitochondrial replacement treatment
Source: EMBO Rep. 2023 Aug 17;24(10):e54540. doi: 10.15252/embr.202154540 (PMC10561356; doi:10.15252/embr.202154540)
Supplement: Supplementary file 1 — Appendix [file EMBR-24-e54540-s006.pdf]

Appendix Figures

Contents:

- **Appendix figure S1 | European mtDNA haplogroup distributions.**
- **Appendix figure S2 | Proportion of women per 100,000 per haplogroup.**
- **Appendix figure S3 | UK egg donors by reported ethnicity.**
- **Appendix figure S4 | mtDNA haplogroup diversity versus estimated population frequency.**
- **Appendix figure S5 | mtDNA haplogroup diversity versus estimated evolutionary age.**
- **Appendix figure S6 | Total mtDNA variant sequence divergence within European, African, and Eurasian mtDNA haplogroup subclades.**
- **Appendix table S1 | Mean mtDNA variant differences between unmatched and haplogroup matched sequence pairs.**

Appendix figure S1

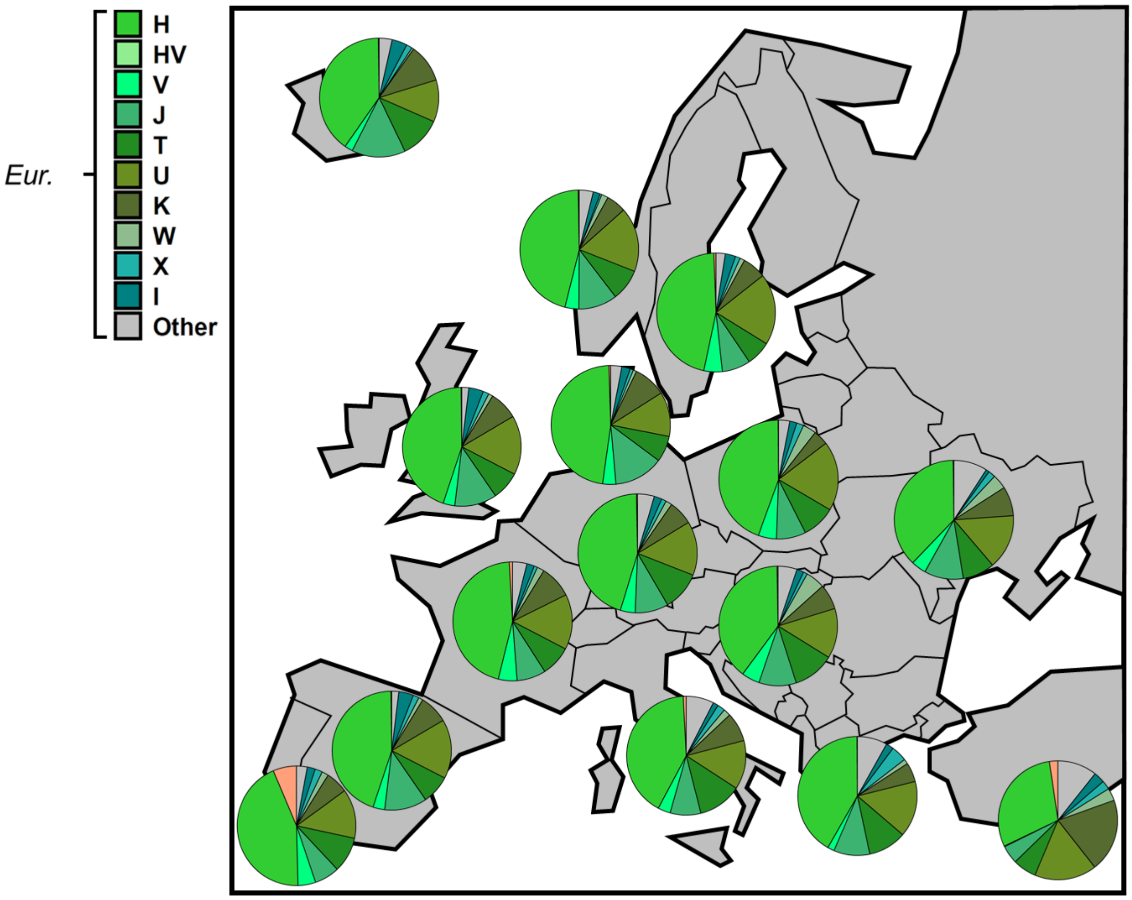

Appendix figure S1 | European mtDNA haplogroup distributions.
Graphical representation of the relative frequency of the 10 major European (Eur) mtDNA haplogroups, which are most likely to contribute to a local donor pool (haplogroups H, HV, V, J, T, U, K, I, W and X), per country (**Dataset EV1a**). In this instance, due to low proportional frequency, 'Other' includes both African and Asian haplogroups).

Appendix figure S2

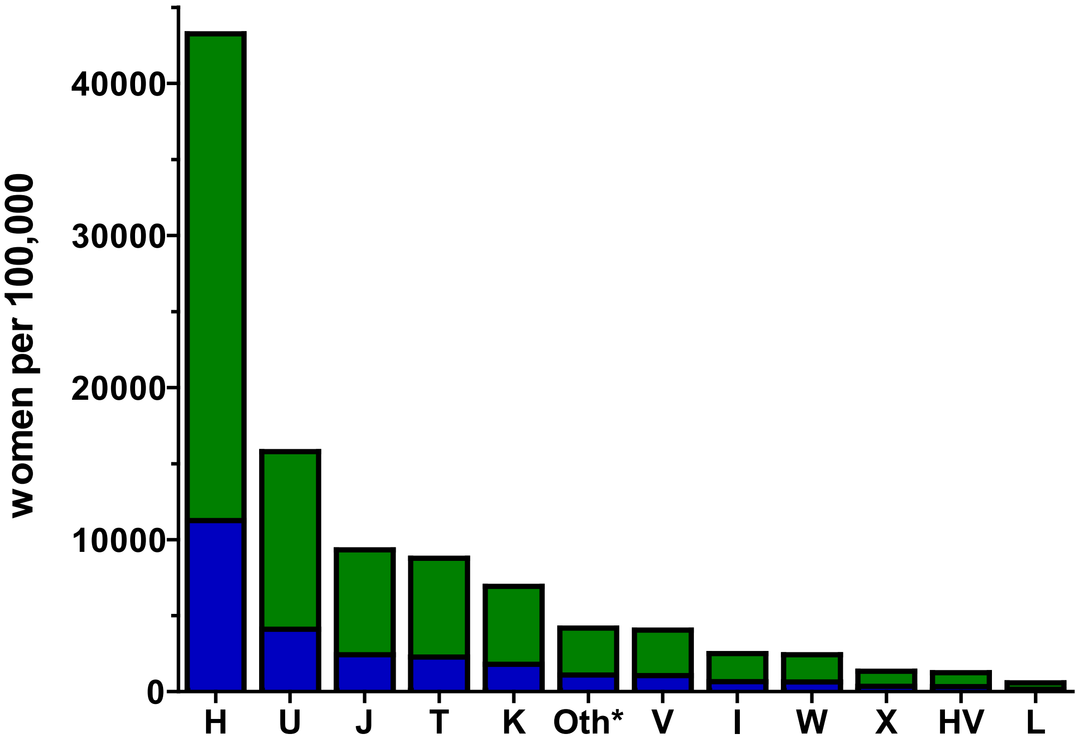

Appendix figure S2 | Proportion of women per 100,000 per haplogroup.
Graph showing the estimated proportion of women per 100,000 in each of the major mtDNA haplogroups in the European population (green) and the proportion of those who would be within the typical age range for egg donation (18-36 years, in blue) (**Dataset EV2a**).

Appendix figure S3

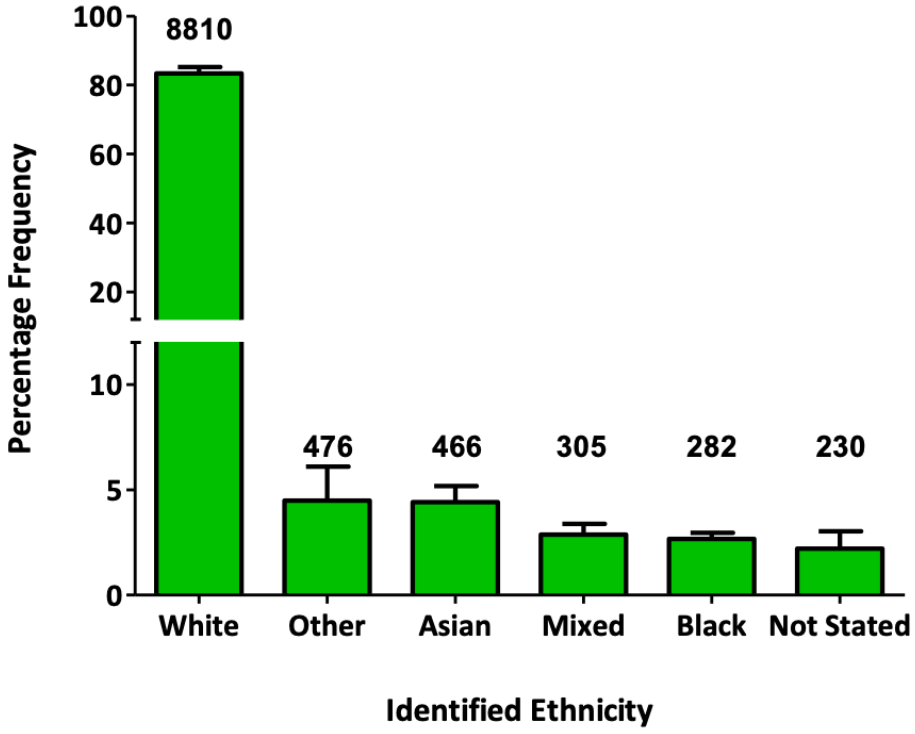

Appendix figure S3 | UK egg donors by reported ethnicity.
Bar graph showing the mean proportion and 95% CI (whiskers) of UK egg donor ethnicities ranked by frequency. The mean and confidence interval represent egg donations over a 5-year period (**Dataset EV2b**). The numbers indicate total number of egg donors in each group obtained from the HFEA report, "HFEA key donor information update 2017."

Appendix figure S4

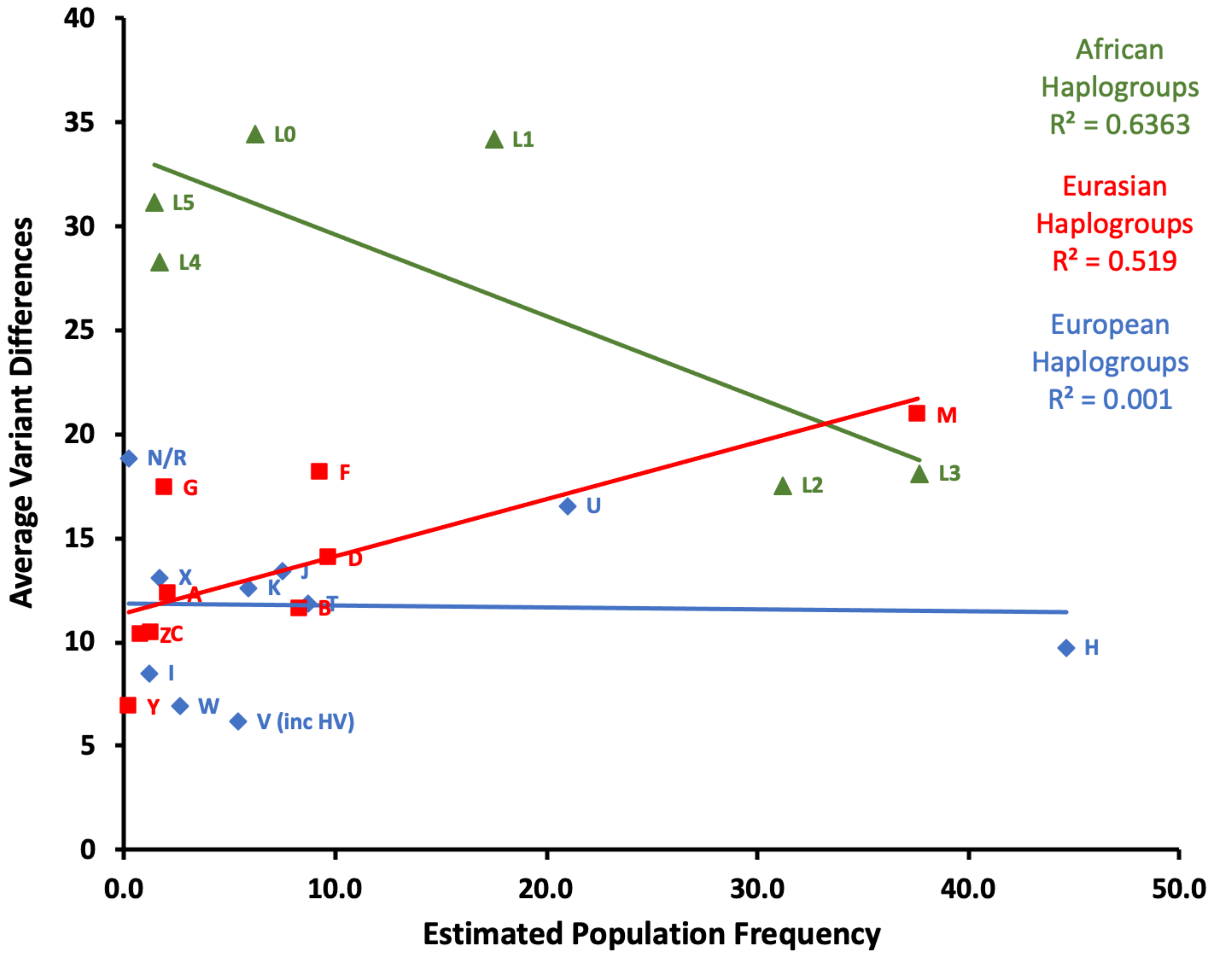

Appendix figure S4 | mtDNA haplogroup diversity versus estimated population frequency. Plot of the average variant differences (y) of the major haplogroups in each population group (African, European and Eurasian, **Datasets EV3a-c**) versus estimated population frequency (y). Shown is the correlation coefficient (R^2).

<u>Appendix figure S5</u>

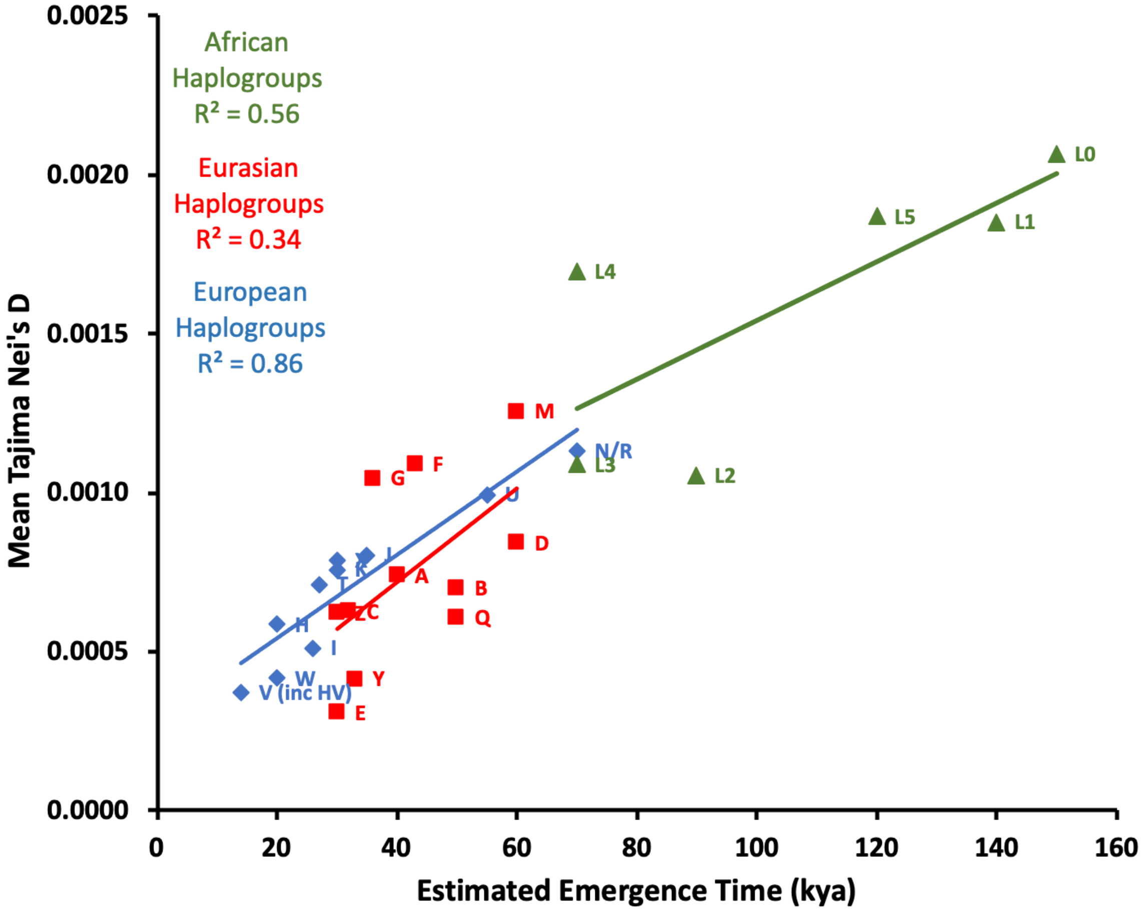

Appendix figure S5 | mtDNA haplogroup diversity versus estimated evolutionary age. Plot of the estimated evolutionary age (x) of the major haplogroups in each population group (African, European and Eurasian, **Datasets EV3a-c**) versus mean Tajima Nei's distance estimates for randomly selected pairs of sequences (y). Shown is the correlation coefficient (R^2).

<u>**Appendix figure S6**</u>

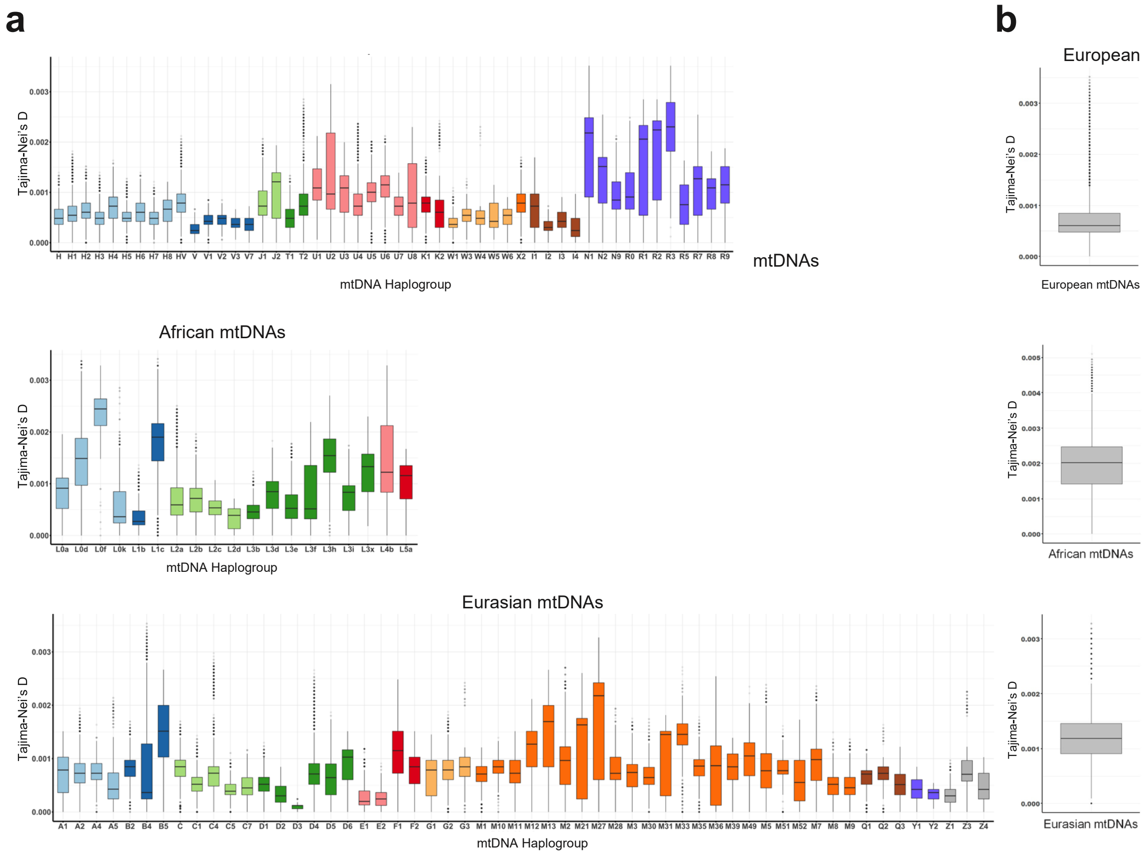

Appendix figure S6 | MtDNA sequence divergence within common European, African and Eurasian haplogroup sub-clades.

a, Boxplots showing estimated mtDNA pairwise sequence divergence within the commonest haplogroup sub-clades of each study population, where upper=European, middle=African and lower=Eurasian (**Datasets EV3a-c**). The intra-haplogroup mtDNA sequence divergence of each population differs significantly (one-way ANOVA in each population was p<2.2x10-16). **b,** Boxplots showing estimated mtDNA pairwise sequence divergence of any two random sequences from within each study population (upper=European, middle=African and lower=Eurasian, **Datasets EV3a-c**). All boxplots show median, 25th and 75th percentile, with whiskers indicating 1.5 upper and lower interquartile range. Dots indicate outliers. Data are based 7,655 European, 3,688 African and 6,857 Eurasian mtDNA sequences. Population groups were defined by mtDNA haplogroup (**Datasets EV3a-c**). Tajima-Nei's genetic distance model (Tajima-Nei's D) was used to investigate sequence divergence (SMaterials), where Tajima-Nei's D 0.00006=1 variant difference.

Appendix table S1| Mean mtDNA variant differences between unmatched and haplogroup matched sequence pairs.
a) Table showing the mean number of variant differences (with 95% CI) between randomly selected unmatched European, African and Eurasian mtDNA pairs (greyed) and the equivalent when sequence pairs are selected from within mtDNA haplogroup subclades in each population (**Datasets EV3a-c**). Arrows indicate either an intra-haplogroup subclade increase or decrease in variant differences relative to unmatched mtDNAs and P is the comparison of intra-haplogroup matched versus unmatched by Mann Whitney U, dashes indicate no significant change. Also shown is the number of sequences (and percentage frequency) used to make the divergence estimates and the maximum number variant differences observed between randomly paired sequences in each haplogroup sub-clade. b) Table showing the mean number of non-synonymous variant differences (with 95% CI) between randomly selected unmatched mtDNA from all datasets (African, European and Eurasian combined) and paired permutations of population group (i.e. African and European mtDNAs, **Datasets EV3d**). Nei-Gojobori model was used to investigate non-synonymous variant differences.

a)

			**intra-group diversity**					
		**% Frequency within dataset**	**No. of sequences for divergence estimate**	**% of dataset**	**Mean Variant Difference (95% CI)**	**Increased (▲) decreased (▼) or not significantly changed (-) compared to total dataset**	**Matched versus Total Dataset P**	**Maximum Variant Differences**
	Total European Dataset	-	7655	-	11.28 (11.16-11.40)	-	-	59
**Major European Haplogroup Subclades**	*H*	*1.4%*	*110*	1.4%	8.92 (8.24-9.60)	▼	*3.3E-08*	*23.20*
	H1	*18.0%*	*1378*	18.0%	9.8 (9.60-9.99)	▼	*4.1E-34*	*30.30*
	H2	*4.4%*	*339*	4.4%	17.24 (11.87-22.60)	-	*1.0E+00*	*39.00*
	H3	*5.5%*	*420*	5.5%	8.15 (7.87-8.44)	▲	*9.7E-67*	*23.25*
	H4	*2.2%*	*166*	2.2%	11.85 (11.14-12.57)	-	*1.0E+00*	*27.23*
	H5	*4.8%*	*366*	4.8%	8.6 (8.29-8.90)	▲	*2.3E-45*	*21.18*
	H6	*2.3%*	*179*	2.3%	10.12 (9.51-10.72)	▼	*1.4E-02*	*23.20*
	H7	*1.6%*	*125*	1.6%	7.86 (7.37-8.35)	▲	*8.8E-25*	*20.17*
	H8	*0.4%*	*30*	0.4%	10.65 (9.03-12.27)	-	*1.0E+00*	*20.18*
	HV	*3.0%*	*228*	3.0%	13.5 (12.86-14.15)	▲	*8.9E-09*	*35.33*
	V	*0.8%*	*61*	0.8%	4.27 (3.72-4.81)	▼	*3.4E-33*	*11.08*

	V1	*1.0%*	*75*	1.0%	7.36 (6.72-7.99)	▼	*1.7E-17*	*14.12*
	V2	*0.3%*	*21*	0.3%	7.56 (6.35-8.78)	▼	*3.5E-04*	*14.12*
	V3	*0.2%*	*17*	0.2%	6.1 (5.06-7.15)	▼	*1.8E-06*	*11.08*
	V7	*0.2%*	*18*	0.2%	6.16 (4.84-7.47)	▼	*3.3E-05*	*12.10*
	J1	*5.9%*	*453*	5.9%	13.26 (12.74-13.77)	▲	*4.3E-11*	*34.38*
	J2	*1.4%*	*109*	1.4%	15.93 (14.41-17.46)	▲	*1.5E-06*	*32.28*
	T1	*2.6%*	*197*	2.6%	8.32 (7.78-8.85)	▼	*1.6E-19*	*22.18*
	T2	*6.2%*	*478*	6.2%	12.45 (12.00-12.91)	▲	*6.7E-05*	*47.50*
	U1	*0.4%*	*32*	0.4%	19.18 (16.86-21.49)	▲	*8.5E-06*	*35.32*
	U2	*1.6%*	*120*	1.6%	22.15 (19.78-24.52)	▲	*2.5E-13*	*52.53*
	U3	*0.6%*	*48*	0.6%	16.6 (14.51-18.69)	▲	*4.4E-04*	*33.28*
	U4	*2.6%*	*198*	2.6%	13.74 (12.69-14.80)	▲	*4.7E-04*	*39.38*
	U5	*7.9%*	*608*	7.9%	16.44 (16.03-16.86)	▲	*8.3E-90*	*33.45*
	U6	*2.7%*	*209*	2.7%	18.32 (17.54-19.09)	▲	*8.6E-42*	*35.32*
	U7	*0.4%*	*31*	0.4%	11.61 (9.87-13.34)	-	*1.0E+00*	*23.18*
	U8	*0.5%*	*39*	0.5%	15.53 (12.05-19.01)	-	*1.0E+00*	*38.33*
	K1	*7.0%*	*539*	7.0%	12.74 (12.40-13.08)	▲	*7.0E-13*	*28.27*
	K2	*1.5%*	*114*	1.5%	10.02 (8.93-11.10)	-	*1.0E+00*	*40.38*
	W1	*0.9%*	*71*	0.9%	6.38 (5.64-7.12)	▼	*1.2E-18*	*18.15*
	W3	*0.3%*	*24*	0.3%	9.52 (8.01-11.02)	-	*1.0E+00*	*21.17*
	W4	*0.1%*	*11*	0.1%	12.53 (5.75-19.30)	-	*1.0E+00*	*38.37*
	W5	*0.2%*	*16*	0.2%	8.41 (6.05-10.76)	-	*1.0E+00*	*19.15*
	W6	*0.2%*	*13*	0.2%	8.76 (6.98-10.54)	-	*8.4E-01*	*15.12*
	X2	*1.9%*	*142*	1.9%	13.11 (12.30-13.93)	▲	*1.2E-03*	*28.25*
	I1	*0.6%*	*45*	0.6%	11.01 (9.23-12.79)	-	*3.8E+01*	*28.25*

	I2	0.5%	36	0.5%	5.57 (4.84-6.29)	▼	8.8E-16	12.10
	I3	0.2%	18	0.2%	7.26 (5.76-8.75)	▼	3.1E-03	15.12
	I4	0.2%	16	0.2%	5.12 (3.37-6.87)	▼	2.5E-04	16.13
	N1	1.1%	82	1.1%	30.48 (27.37-33.59)	▲	4.1E-18	58.65
	N2	0.3%	22	0.3%	21.44 (17.12-25.76)	▲	7.6E-03	42.43
	N9	1.7%	129	1.7%	15.1 (13.86-16.34)	▲	9.2E-07	35.33
	R0	1.5%	116	1.5%	17.26 (15.76-18.75)	▲	1.4E-10	41.42
	R1	0.4%	30	0.4%	26.06 (20.70-31.43)	▲	4.1E-04	47.47
	R2	0.3%	23	0.3%	29.08 (22.88-35.29)	▲	5.9E-04	47.48
	R3	0.3%	20	0.3%	36.98 (31.74-42.23)	▲	5.0E-07	58.65
	R5	0.3%	25	0.3%	12.86 (10.11-15.60)	-	1.0E+00	27.23
	R7	0.2%	18	0.2%	18.38 (13.28-23.48)	-	7.2E-01	42.42
	R8	0.8%	65	0.8%	16.67 (15.17-18.17)	▲	7.7E-08	30.27
	R9	0.3%	25	0.3%	18.6 (15.65-21.55)	▲	3.0E-03	31.28

**Major African Haplogroup Subclades**	Total African Dataset	-	3688	-	32.80 (32.33-33.27)	-	-	85
	L0a	8.6%	318	8.6%	13.29 (12.61-13.97)	▼	1.2E-197	32.63
	L0d	14.7%	543	14.7%	23.68 (22.74-24.61)	▼	1.9E-51	56.17
	L0f	0.4%	16	0.4%	37.78 (31.86-43.70)	-	1.0E+00	54.75
	L0k	2.9%	106	2.9%	9.23 (7.73-10.73)	▼	6.8E-55	47.53
	L1b	6.9%	255	6.9%	5.77 (5.34-6.20)	▼	4.5E-16	22.65
	L1c	12.2%	449	12.2%	28.82 (27.84-29.81)	▼	7.8E-09	56.87
	L2a	17.2%	636	17.2%	11.71 (11.20-12.23)	▼	3.8E-16	41.75

	L2b	2.1%	79	2.1%	11.06 (9.78-12.33)	▼	4.7E-50	32.63
	L2c	2.8%	104	2.8%	8.77 (8.17-9.36)	▼	3.1E-141	17.85
	L2d	0.4%	16	0.4%	5.84 (4.31-7.37)	▼	1.3E-15	11.88
	L3b	3.9%	142	3.9%	7.41 (6.91-7.92)	▼	4.3E-223	20.60
	L3d	5.7%	212	5.7%	12.66 (11.75-13.56)	▼	3.1E-118	30.48
	L3e	13.5%	497	13.5%	9.34 (8.87-9.81)	▼	6.6E-15	29.53
	L3f	4.3%	160	4.3%	13.11 (11.63-14.58)	▼	1.3E-58	36.53
	L3h	1.2%	44	1.2%	25.06 (22.41-27.72)	▼	7.2E-05	45.07
	L3i	0.4%	14	0.38%	13.11 (9.35-16.87)	▼	3.1E-06	28.93
	L3x	0.6%	21	0.57%	20.52 (17.30-23.74)	▼	1.1E-05	38.32
	*L4b**	0.9%	33	0.9%	22.77 (18.23-27.31)	▼	5.1E-03	54.77
	*L5a***	0.7%	27	0.7%	16.79 (14.02-19.55)	▼	4.3E-10	27.87

**Major Eurasian Haplogroup Subclades**	Total Eurasian Dataset	-	6857	-	20.12 (19.95-20.28)	-	-	59
	A1	0.2%	14	0.2%	12.02 (8.83-15.22)	▼	1.4E-02	25.20
	A2	4.1%	281	4.1%	12.51 (11.96-13.06)	▼	2.5E-80	32.32
	A4	0.8%	56	0.8%	12.31 (11.31-13.30)	▼	4.1E-20	27.25
	A5	1.0%	66	1.0%	8.59 (7.09-10.08)	▼	2.6E-21	35.67
	B2	2.0%	136	2.0%	13.81 (13.18-14.44)	▼	8.7E-40	28.27
	B4	11.6%	792	11.6%	11.49 (10.78-12.19)	▼	6.6E-93	58.87
	B5	1.0%	66	1.0%	24.72 (22.29-27.16)	▲	2.5E-02	44.45
	C	2.2%	153	2.2%	13.32 (12.48-14.16)	▼	2.1E-32	28.30
	C1	3.2%	220	3.2%	8.68 (8.26-9.09)	▼	2.7E-141	23.83

	C4	5.6%	382	5.6%	11.34 (10.83-11.85)	▼	8.5E-118	49.62
	C5	1.5%	105	1.5%	6.65 (6.07-7.24)	▼	4.7E-73	18.40
	C7	0.8%	57	0.8%	8.03 (7.08-8.98)	▼	8.7E-31	19.30
	D1	3.6%	250	3.6%	8.61 (8.21-9.00)	▼	1.2E-163	21.63
	D2	0.9%	62	0.9%	5.42 (4.58-6.27)	▼	3.5E-41	14.10
	D3	0.4%	26	0.4%	1.66 (1.26-2.06)	▼	1.7E-40	4.03
	D4	14.0%	963	14.0%	12.26 (11.95-12.57)	▼	7.3E-274	44.30
	D5	3.2%	216	3.2%	10.48 (9.69-11.27)	▼	1.4E-60	31.12
	D6	0.4%	24	0.4%	14.98 (12.52-17.43)	▼	2.3E-02	25.22
	E1	2.5%	169	2.5%	4.75 (4.22-5.28)	▼	6.5E-121	19.70
	E2	0.9%	65	0.9%	4.31 (3.68-4.94)	▼	9.1E-55	15.17
	F1	1.9%	131	1.9%	18.26 (16.93-19.60)	-	4.3E-01	41.40
	F2	0.2%	17	0.2%	12.9 (10.23-15.58)	▼	3.9E-03	25.22
	G1	1.2%	82	1.2%	11.17 (9.87-12.47)	▼	8.9E-21	24.20
	G2	1.9%	132	1.9%	13.35 (12.49-14.21)	▼	6.2E-30	31.30
	G3	0.4%	25	0.4%	14.98 (11.88-18.09)	-	1.9E-01	40.35
	M1	1.8%	125	1.8%	11.56 (10.90-12.21)	▼	6.2E-52	20.15
	M10	0.5%	32	0.5%	13.83 (12.43-15.24)	▼	4.4E-08	23.18
	M11	0.4%	28	0.4%	12.84 (11.02-14.66)	▼	1.0E-06	25.22
	M12	0.6%	42	0.6%	19.25 (16.64-21.87)	-	1.0E+00	35.30
	M13	0.6%	40	0.6%	23.4 (19.73-27.07)	-	1.0E+00	44.40
	M2	2.3%	160	2.3%	15.8 (14.48-17.12)	▼	9.7E-08	45.08
	M21	0.6%	38	0.6%	20.21 (16.16-24.26)	-	1.0E+00	43.40
	M27	2.1%	142	2.1%	27.85 (25.15-30.54)	▲	5.8E-06	54.53
	M28	0.7%	51	0.7%	12.96 (11.26-14.66)	▼	3.9E-09	32.27

	M3	1.9%	129	1.9%	11.52 (10.58-12.47)	▼	7.3E-35	29.18
	M30	1.1%	73	1.1%	11.06 (10.07-12.05)	▼	5.1E-27	29.02
	M31	0.4%	27	0.4%	17.51 (13.54-21.48)	-	1.0E+00	30.27
	M33	0.7%	50	0.7%	23.11 (20.97-25.24)	-	4.8E-01	45.23
	M35	0.8%	56	0.8%	13.48 (12.18-14.77)	▼	1.8E-12	29.68
	M36	0.6%	38	0.6%	12.06 (8.64-15.48)	▼	2.7E-03	42.40
	M39	0.4%	27	0.4%	13.91 (11.81-16.00)	▼	2.6E-04	23.17
	M49	0.4%	30	0.4%	16.18 (12.85-19.50)	-	1.0E+00	39.15
	M5	2.1%	144	2.1%	13.59 (12.39-14.79)	▼	3.1E-18	39.73
	M51	0.4%	26	0.4%	12.73 (9.99-15.47)	▼	9.5E-04	26.85
	M52	0.5%	37	0.5%	10.14 (7.74-12.53)	▼	5.1E-08	28.83
	M7	7.9%	542	7.9%	14.48 (13.91-15.04)	▼	2.1E-61	39.30
	M8	0.6%	44	0.6%	8.49 (7.24-9.74)	▼	1.7E-20	24.87
	M9	2.6%	180	2.6%	8.13 (7.60-8.67)	▼	3.4E-105	22.70
	Q1	1.6%	113	1.6%	10.33 (9.50-11.17)	▼	1.2E-43	19.38
	Q2	0.4%	26	0.4%	11.75 (9.53-13.98)	▼	5.7E-06	27.22
	Q3	0.2%	17	0.2%	8.56 (6.42-10.70)	▼	6.4E-07	20.35
	Y1	0.3%	20	0.3%	7.37 (5.92-8.82)	▼	1.8E-11	14.12
	Y2	0.2%	13	0.2%	5.74 (4.57-6.90)	▼	3.5E-10	9.07
	Z1	1.0%	66	1.0%	4.93 (4.25-5.61)	▼	1.2E-51	16.12
	Z3	0.5%	36	0.5%	13.41 (10.71-16.11)	▼	1.4E-03	37.55
	Z4	0.2%	15	0.2%	7.79 (5.52-10.07)	▼	2.3E-06	17.13

b)

African, European and Eurasian mtDNAs	-	18,200	-	19.64 (19.5-19.9)	-	-	85
European and African mtDNAs	-	11,343	63.3%	24.92 (24.7-25.2)	▲	3.8E-302	85
European and Eurasian mtDNAs	-	14,512	79.7%	18.22 (18.1-18.3)	▼	4.9E-46	55
African and Eurasian mtDNAs	-	10,454	57.4%	23.76 (23.6-24.0)	▲	5.6E-298	80
