# Supplementary material for: Feasibility and impact of haplogroup matching for mitochondrial replacement treatment
Source: EMBO Rep. 2023 Aug 17;24(10):e54540. doi: 10.15252/embr.202154540 (PMC10561356; doi:10.15252/embr.202154540)
Supplement: Supplementary file 2 — Expanded View Figures PDF [file EMBR-24-e54540-s004.pdf]

# Expanded View Figures

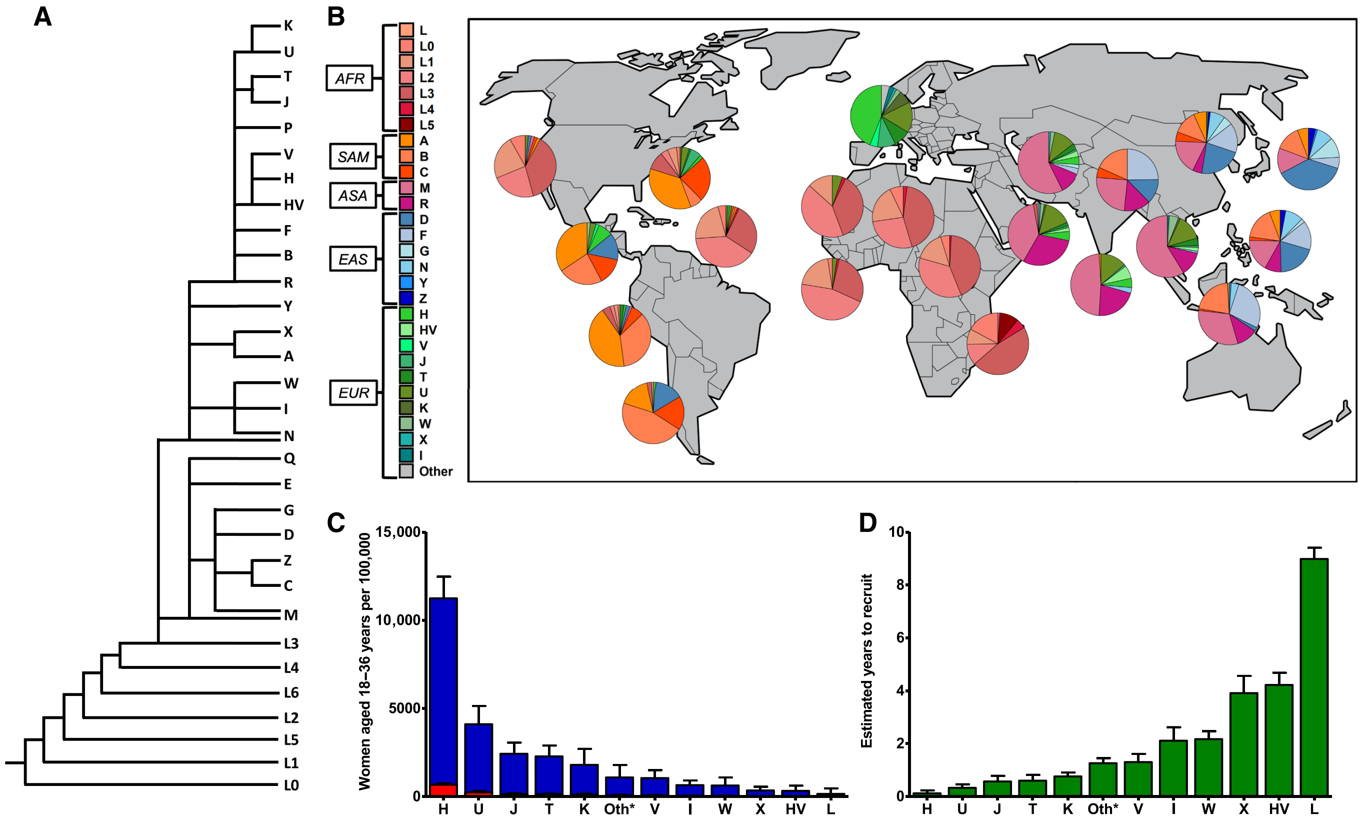


**Figure EV1. Impact of haplogroup matching on the availability of egg donors for MRT.**

A Simplified mtDNA haplogroup phylogeny showing the 31 'major' mtDNA haplogroups (phylotree.org).

B Graphical representation of global mtDNA haplogroup diversity, showing the relative estimated proportions of the most frequent mtDNA haplogroups by region (where available, AFR = Africa, SAM = South America, ASA = Southern Asia, EAS = East Asia and EUR = Europe, Dataset EV1A and B).

C Graph showing the estimated number of women per 100,000 that proceed to egg donation (red) in relation to those that are eligible to donate (blue) for each of the commonest mtDNA haplogroups identified across European populations (Based on 316 donors, Datasets EV1A and EV2A, and assuming the haplogroup of those who donate eggs is reflective of ancestry).

D Estimated years to recruit one volunteer per European mtDNA haplogroup based on the estimated donor availability and the estimated number of women who progress to egg donation (Datasets EV1A and EV2A).

Data information: Bar graphs show mean and standard deviation.


© 2023 The Authors
