# Supplementary material for: Feasibility and impact of haplogroup matching for mitochondrial replacement treatment
Source: EMBO Rep. 2023 Aug 17;24(10):e54540. doi: 10.15252/embr.202154540 (PMC10561356; doi:10.15252/embr.202154540)
Supplement: Supplementary file 7 — PDF+ [file EMBR-24-e54540-s007.pdf]

Exploratory Report

TRANSPARENT PROCESS OPEN ACCESS

EMBO reports

Feasibility and impact of haplogroup matching for mitochondrial replacement treatment

Yuko Takeda[1], Louise Hyslop[2], Meenakshi Choudhary[2], Fiona Robertson[3], Angela Pyle[3], Ian Wilson[4], Mauro Santibanez-Koref[4], Douglass Turnbull[3], Mary Herbert[1,2,5,*] & Gavin Hudson[1,**]

Abstract

Mitochondrial replacement technology (MRT) aims to reduce the risk of serious disease in children born to women who carry pathogenic mitochondrial DNA (mtDNA) variants. By transplanting nuclear genomes from eggs of an affected woman to enucleated eggs from an unaffected donor, MRT creates new combinations of nuclear and mtDNA. Based on sets of shared sequence variants, mtDNA is classified into ~30 haplogroups. Haplogroup matching between egg donors and women undergoing MRT has been proposed as a means of reducing mtDNA sequence divergence between them. Here we investigate the potential effect of mtDNA haplogroup matching on clinical delivery of MRT and on mtDNA sequence divergence between donor/recipient pairs. Our findings indicate that haplogroup matching would limit the availability of egg donors such that women belonging to rare haplogroups may have to wait > 4 years for treatment. Moreover, we find that intra-haplogroup sequence variation is frequently within the range observed between randomly matched mtDNA pairs. We conclude that haplogroup matching would restrict the availability of MRT, without necessarily reducing mtDNA sequence divergence between donor/recipient pairs.

Keywords haplogroup matching; mitochondrial disease; mitochondrial replacement therapy
Subject Categories Genetics, Gene Therapy & Genetic Disease
DOI 10.15252/embr.202154540 | Received 17 December 2021 | Revised 3 July 2023 | Accepted 26 July 2023 | Published online 17 August 2023
EMBO Reports (2023) 24: e54540

Introduction

Mitochondrial DNA (mtDNA) is maternally inherited and encodes 37 genes, including 13 proteins, which together with nuclear-encoded proteins generate ~90% of cellular ATP by oxidative phosphorylation (Chinnery & Hudson, 2013). Pathogenic variants in mtDNA cause a broad spectrum of life-limiting conditions estimated to affect 1 in 5,000 adults (Gorman *et al*, 2016). In the absence of effective curative treatments for the majority of conditions, there has been a growing interest in the development of assisted reproductive technologies (ART) to prevent transmission of mtDNA disease.

Preimplantation Genetic Testing (PGT) is a well-established ART procedure for detecting defects in the nuclear genome and has been applied successfully to detect pathogenic mtDNA variants in preimplantation embryos (Smeets *et al*, 2015). PGT can reduce the risk of disease by identifying embryos with low variant loads (Smeets *et al*, 2015). In cases where no low-load embryos are available, mitochondrial replacement technology (MRT) offers the potential to reduce transmission of pathogenic variants, thereby reducing the risk of mtDNA disease. MRT involves transplanting the nuclear genome from affected eggs to an enucleated egg from an unaffected donor which largely replaces mutated mtDNA with wildtype (Hyslop *et al*, 2016; Kang *et al*, 2016; Yamada *et al*, 2016). The procedure has been approved for cautious clinical application in the UK where it is permitted for use solely in cases with a high risk of transmitting serious mtDNA disease, and for which PGT is unsuitable (Greenfield, 2016; Greenfield *et al*, 2017; Herbert *et al*, 2023).

Worldwide, mtDNA can be phylogenetically classified into ~30 different 'haplogroups' based on specific sets of shared, common mtDNA variants (van Oven & Kayser, 2009). MtDNA haplogroups are indicative of maternal continental-ancestry (Emery *et al*, 2015), and whilst human migration has blurred geographical boundaries in haplogroup frequencies, there remain marked differences in haplogroup distributions across different world populations (Fig EV1). In sexually reproducing organisms, each round of meiosis and fertilisation generates new combinations of nuclear and mitochondrial genomes, which in humans may involve diverse ancestries (Wei *et al*, 2019). MRT achieves this by artificial means, raising the question of whether mtDNA sequence divergence between egg donors and patient recipients might influence the outcome of the procedure. MtDNA haplogroup matching has therefore been proposed as a precautionary measure to reduce mtDNA sequence

1 Wellcome Centre for Mitochondrial Research, Biosciences Institute, Newcastle University, Newcastle upon Tyne, UK
2 Newcastle Fertility Centre, Biomedicine West Wing, Centre for Life, Newcastle upon Tyne, UK
3 Wellcome Centre for Mitochondrial Research, Institute of Clinical Translational Research, Newcastle University, Newcastle upon Tyne, UK
4 Biosciences Institute, Centre for Life, Newcastle upon Tyne, UK
5 Department of Anatomy and Developmental Biology, Monash Biomedicine Discovery Institute, Monash University, Melbourne, VIC, Australia
*Corresponding author. Tel: +44 0191 213 8213; E-mail: mary.herbert@ncl.ac.uk
**Corresponding author. Tel: +44 0191 208 5397; E-mail: gavin.hudson@ncl.ac.uk

© 2023 The Authors. Published under the terms of the CC BY 4.0 license

divergence between egg donors and women undergoing MRT (Burgstaller *et al*, 2015; Morrow *et al*, 2015; Latorre-Pellicer *et al*, 2016; Royrvik *et al*, 2016).

The MRT procedure requires highly specialised skills and is therefore likely to be offered in specialist centres and women requiring treatment are likely to be referred from a broad geographical area. Because pathogenic mtDNA mutations are agnostic to mtDNA haplogroup (Ramos *et al*, 2013), the diversity of mtDNA lineages among patients from a broad geographical distribution is likely to exceed that of the egg donor population local to the treating centre. In theory, it might be possible to overcome geographical constraints by importing cryostored (vitrified) donor egg from the relevant phylogeographic regions. However, the scope for this is currently limited owing to reduced efficacy of MRT when vitrified donor eggs are used (Hyslop *et al*, 2016). Thus, current clinical protocols involve the use of freshly harvested donor eggs, donated largely by women local to the clinic.

In view of a general shortage of donated eggs (Platts *et al*, 2021), we investigated the likely impact of haplogroup matching on the availability of donated eggs and on the mtDNA sequence divergence between egg donors and women undergoing MRT. Our findings indicate that women belonging to rare or under-represented mtDNA haplogroups could face a waiting time of > 4 years for a haplogroup matched egg donor. Moreover, analysis of mtDNA sequence variation within and between haplogroups revealed that intra-haplogroup sequence variation is within the range observed for randomly sampled pairs of mtDNA. Notably, intra-haplogroup sequence variation is comparatively higher for some rarer haplogroups. In such cases, women may have to spend a substantial fraction of their reproductive years, waiting for haplogroup matched donor for little or no gain in mtDNA sequence similarity.

Results

Effect of haplogroup frequency on the availability of donated eggs for MRT

The phylogenetic classification of mtDNA into ~30 'major' haplogroups (van Oven & Kayser, 2009; Fig EV1A) and their geographical distribution across different world populations (Fig EV1B) illustrates the requirement for egg donors from across the phylogeny to implement a haplogroup matching strategy in specialist MRT treatment centres.

Taking the European population as an exemplar, H is the predominant major haplogroup with a frequency of ~43% (Appendix Fig S1). Because of the age-related decline in the number and quality of eggs (Herbert & Turnbull, 2015), the typical age range for egg donors is 18–36 years, which corresponds to ~26% of the female population (Appendix Fig S2). In a European context, potential donors aged 18–36 years, for H recipients would be relatively frequent, representing ~11,200 per 100,000 women (~11%, Fig EV1C). However, finding a matched egg donor for women belonging to rare haplogroups would be challenging. For example, for haplogroup X, which is detectable at ~1.4% across Europe (Reidla *et al*, 2003), the pool of potential egg donors represents only ~351 per 100,000 women (~0.35%, Fig EV1C). Haplogroup matching within African and Eurasian populations would be similarly challenging. For example, of the 11 major Eurasian haplogroups, seven occur at an estimated frequency of < 2.1%, which in combination account for ~6% of the population (Table 1). Thus, mtDNA haplogroup matching would universally restrict the availability of MRT for women belonging to rare haplogroups.

Whilst the above estimates are based on the theoretical availability of egg donors, the availability of donated eggs varies widely between different countries (Pennings *et al*, 2014). Analysis of data from our in-house egg donation programme indicates that whereas ~1 in 1,000 of the local female population expresses an interest in donating eggs, only 6% of those who express interest eventually complete a donation cycle. Given the expected frequency of haplogroups in the local population, the number of potential egg donors for patients belonging to rare haplogroups becomes vanishingly small (Fig EV1C). For example, we estimate that women belonging to haplogroup X may have to wait for up to 4 years for a matched donor, compared with ~1 month for women belonging to haplogroup H (Fig EV1D).

In addition to the challenges associated with intrinsically rare haplogroups, the challenge of finding haplogroup matched donors is greatly increased for women seeking treatment in a geographical location that differs from the continental origin of their maternal ancestry. For example, potential egg donors belonging to the African haplogroup L represent just ~153 per 100,000 women in the UK (~0.15% of women, Fig EV1C). This problem is compounded by a lack of diversity among women who donate eggs. Data recorded by the Human Fertilisation and Embryology Authority over the last 5 years indicates that the majority (89%) of women who donate eggs in the UK self-identify as 'white' (Appendix Fig S3) (HFEA, 2023). Based on limited data, egg donors in the US show a similar ethnicity profile (Sachs *et al*, 2010). Since ethnicity can be inferred from mtDNA sequence (~90% accuracy; Lee *et al*, 2011), it can be assumed that the majority of women self-identifying as 'white' belong to a European-origin mtDNA lineages. Based on haplogroup frequencies, we estimate that it could take 9–10 years to recruit a matched egg donor for an MRT patient belonging to haplogroup L (Fig EV1D). Thus, mtDNA haplogroup matching would impose severe restrictions on the provision of MRT to women whose mtDNA lineage is underrepresented among egg donors in the region local to the treating centre.

Is mtDNA sequence divergence reduced by haplogroup matching?

In light of the restrictions, it would impose on the delivery of MRT, we investigated the extent to which haplogroup matching would reduce mtDNA sequence divergence between egg donors and women undergoing MRT. Using Tajima-Nei's genetic distance (D) estimates (Tajima & Nei, 1983), we find considerable mtDNA sequence divergence within haplogroups (Fig 1A). Across European, African, and Eurasian populations, haplogroup matched mtDNA pairs differ by as many as 59 (range = 14–59), 81 (range = 44–81) and 59 (range = 14–59) variants respectively (Table 1). Whilst Eurasian haplogroup frequency is positively correlated with intra-haplogroup sequence divergence, the opposite is true for African haplogroups and European haplogroups show no correlation between haplogroup frequency and sequence divergence (Appendix Fig S4). Across all populations, intra-haplogroup sequence variation is positively correlated with the estimated haplogroup evolutionary age (Soares *et al*, 2009) (Appendix Fig S5). Crucially, sequence

 © 2023 The Authors

Table 1. Mean mtDNA variant differences between unmatched and haplogroup matched sequence pairs.

		Intra-group diversity						
	**Estimated % population frequency (SD)**	**No. of sequences for divergence estimate**	**% of Dataset**	**Mean Tajima-Nei *D* × 10^{-3} (95% CI)**	**Mean Variant Difference (95% CI)**	**Increased (▲) or Decreased (▼) Compared to Total Dataset**	**Matched versus total dataset *P***	**Maximum variant differences**
**A**								
Major European Haplogroups								
Total European Dataset	–	7,655	–	0.68 (0.67–0.68)	11.28 (11.16–11.40)	–	–	59
H	44.6 (± 5.4)	3,113	40.7%	0.58 (0.58–0.59)	9.74 (9.60–9.87)	▼	5.5E-64	35
V (inc HV)	5.4 (± 2.1)	420	5.5%	0.37 (0.35–0.39)	6.19 (5.90–6.47)	▼	6.7E-131	14
J	7.5 (± 2.6)	562	7.3%	0.80 (0.78–0.83)	13.40 (12.92–13.88)	▲	2.5E-15	34
T	8.7 (± 3.6)	675	8.8%	0.71 (0.69–0.73)	11.85 (11.47–12.24)	▲	5.7E-02	48
U	21.0 (± 8.8)	1,285	16.8%	0.99 (0.97–1.01)	16.56 (16.23–16.89)	▲	8.3E-152	53
K	5.9 (± 1.9)	653	8.5%	0.76 (0.74–0.78)	12.62 (12.30–12.95)	▲	5.3E-13	40
W	2.6 (± 1.9)	135	1.8%	0.42 (0.38–0.45)	6.92 (6.27–7.57)	▼	7.4E-25	38
X	1.7 (± 2.3)	142	1.9%	0.79 (0.74–0.84)	13.11 (12.30–13.93)	▲	2.4E-04	28
I	1.2 (± 0.9)	115	1.5%	0.51 (0.45–0.57)	8.52 (7.52–9.52)	▼	3.6E-06	28
N/R	0.2 (± 0.4)	555	7.3%	1.13 (1.08–1.19)	18.86 (17.96–19.77)	▲	2.1E-48	**59**
Major African Haplogroups								
Total African Dataset	–	3,688	–	1.97 (1.94–2.00)	32.80 (32.33–33.27)	–	–	85
L0	6.2 (± 5.6)	983	26.7%	2.07 (2.01–2.12)	34.45 (33.53–35.37)	▲	1.3E-04	81
L1	17.5 (± 5.3)	704	19.1%	2.05 (1.94–2.16)	34.20 (32.37–36.03)	▲	5.7E-02	58
L2	32.1 (± 12.3)	835	22.6%	1.05 (1.01–1.09)	17.55 (16.85–18.24)	▼	3.1E-202	61
L3	37.7 (± 7.7)	1,090	29.6%	1.09 (1.06–1.11)	18.13 (17.67–18.58)	▼	1.2E-16	44
L4	1.7 (± 1.6)	42	1.1%	1.70 (1.45–1.94)	28.28 (24.21–32.35)	▼	3.8E-01	55
L5	1.4 (± 3.8)	34	0.9%	1.87 (1.44–2.30)	31.14 (24.02–38.26)	▼	1.0E+00	69
Major Eurasian Haplogroups								
Total Eurasian Dataset	–	6,857	–	1.21 (1.20–1.22)	20.12 (19.95–20.28)	–	–	59
A	2.1 (± 2.8)	417	6.1%	0.74 (0.71–0.77)	12.31 (11.85–12.77)	▼	2.7E-121	36
B	8.3 (± 2.8)	994	14.5%	0.70 (0.66–0.74)	11.64 (11.01–12.27)	▼	1.7E-113	**59**
C	1.3 (± 2.1)	917	13.4%	0.63 (0.61–0.64)	10.42 (10.16–10.69)	▼	4.2E-33	38
D	9.7 (± 10.6)	1,541	22.5%	0.84 (0.83–0.86)	14.03 (13.76–14.30)	▼	1.2E-252	42
E	n.a.	234	3.4%	0.31 (0.28–0.33)	5.12 (4.71–5.53)	▼	1.3E-185	20
F	9.3 (± 10.6)	148	2.2%	1.09 (1.02–1.17)	18.18 (16.93–19.43)	▼	3.5E-02	41
G	1.9 (± 3.4)	239	3.5%	1.05 (1.00–1.09)	17.43 (16.64–18.22)	▼	3.1E-09	44
M	37.6 (± 19.5)	2,061	30.1%	1.26 (1.24–1.27)	20.94 (20.64–21.23)	▲	2.0E-05	53
Q	n.a.	156	2.3%	0.61 (0.55–0.67)	10.11 (9.11–11.11)	▼	6.7E-43	38
Y	0.2 (± 0.4)	33	0.5%	0.41 (0.35–0.48)	6.89 (5.83–7.95)	▼	8.6E-22	14
Z	0.8 (± 1.4)	117	1.7%	0.62 (0.55–0.69)	10.33 (9.17–11.50)	▼	2.0E-31	38

© 2023 The Authors

Table 1 (continued)

	Estimated % population frequency (SD)	Intra-group diversity: No. of sequences for divergence estimate	% of Dataset	Mean Tajima-Nei $D \times 10^{-3}$ (95% CI)	Mean Variant Difference (95% CI)	Increased (▲) or Decreased (▼) Compared to Total Dataset	Matched versus total dataset P	Maximum variant differences
**B**								
African, European and Eurasian mtDNAs	–	18,200	–	1.18 (1.17–1.19)	19.64 (19.5–19.9)	–	–	85
European and African mtDNAs	–	11,343	63.3%	1.50 (1.49–1.50)	24.92 (24.7–25.2)	▲	3.8E-302	85
European and Eurasian mtDNAs	–	14,512	79.7%	1.09 (1.02–1.17)	18.22 (18.1–18.3)	▼	4.9E-46	55
African and Eurasian mtDNAs	–	10,454	57.4%	1.43 (1.44)	23.76 (23.6–24.0)	▲	5.6E-298	80

(A) Mean Tajima-Nei distances (with 95% CI) and equivalent mean number of variant differences (with 95% CI) between randomly selected unmatched European, African, and Eurasian mtDNA pairs (greyed) and when sequence pairs are selected from within mtDNA haplogroups (Dataset EV3A–C). Arrows indicate either an intra-haplogroup increase or decrease in variant differences relative to unmatched mtDNAs and P is the comparison of intra-haplogroup matched versus unmatched by Mann–Whitney U, dashes indicate no significant change. Shown is the number of sequences (and percentage frequency) used to make the divergence estimates and the maximum number variant differences observed between randomly paired sequences in each haplogroup. Population frequency is taken from phase 31,000 Genomes data is included (Dataset EV1A and B, where n.a. is not available, European = 503, African = 660 and Eurasian = 993 mtDNAs). (B) Mean Tajima-Nei distances (with 95% CI) and equivalent mean number of variant differences (with 95% CI) between randomly selected unmatched mtDNA from all datasets (African, European and Eurasian combined) and paired permutations of population group (e.g., African and European mtDNAs, Dataset EV3D).

diversity within some haplogroups either exceeds, or is similar to that observed between randomly sampled pairs of mtDNA from the combined major haplogroups within each population (Fig 1A and B and Table 1).

A comparison of the differences in the mean variant count of haplogroup matched and randomly sampled mtDNA pairs indicates that haplogroup matching would result in a reduction in the mean variant count (ranging from ~1.5 to 5.0 variants) in four of the 10 European-origin major haplogroups, including H, which is the most prevalent (Fig 1C). By contrast, the mean variant count of the six less prevalent European origin haplogroups would be increased by 0.6–7.6 variants by haplogroup matching. Among African- and Eurasian-origin haplogroups, the mean variant difference between haplogroup matched pairs is, in most cases (5/6 African and 14/17 Eurasian major haplogroups) reduced by 1.4–14.7 and 1.9–15.0 variants respectively, compared with randomly sampled pairs within each population (Fig 1C).

In summary, MRT involving donor/patient pairs from the same population, matching according to major mtDNA haplogroups has the potential to reduce sequence divergence within African and Eurasian mtDNA lineages. However, for the majority of European haplogroups, particularly rarer haplogroups, the average variant difference could potentially be reduced by randomly selecting an egg donor rather than waiting, possibly for years, for a haplogroup-matched donor.

Variation in non-synonymous haplogroup-defining mtDNA sequences

The majority of common (~73%) of haplogroup-defining variants are synonymous (Elson *et al*, 2004; Wei *et al*, 2017) and are unlikely to modulate mitochondrial function. Conversely, common non-synonymous variants are likely to impact mitochondrial function (Gomez-Duran *et al*, 2012; Cai *et al*, 2021) and natural selection has shaped mtDNA, resulting in haplogroups with different non-synonymous variant counts (Elson *et al*, 2004; Wei *et al*, 2017). We therefore asked whether haplogroup matching might reduce the number of non-synonymous variant differences between donors and MRT recipients.

Our findings indicate that the overall pattern of variation within haplogroups is similar between total and non-synonymous sequence variants (Figs 1A and 2A and B). However, in some cases (6/27), particularly among European haplogroups, the mean non-synonymous variant count would be increased by haplogroup matching donor/patient pairs (Fig 2C and Table 2). Strikingly, among Eurasian haplogroups, the benefit of haplogroup matching (Fig 1C) is largely lost for non-synonymous variants (Fig 2C). Thus, for non-synonymous variants, which are more likely to have a functional relevance, the mean variant difference would, in most cases, be either increased or unchanged by haplogroup matching egg donors with women undergoing MRT (Table 2).

 © 2023 The Authors

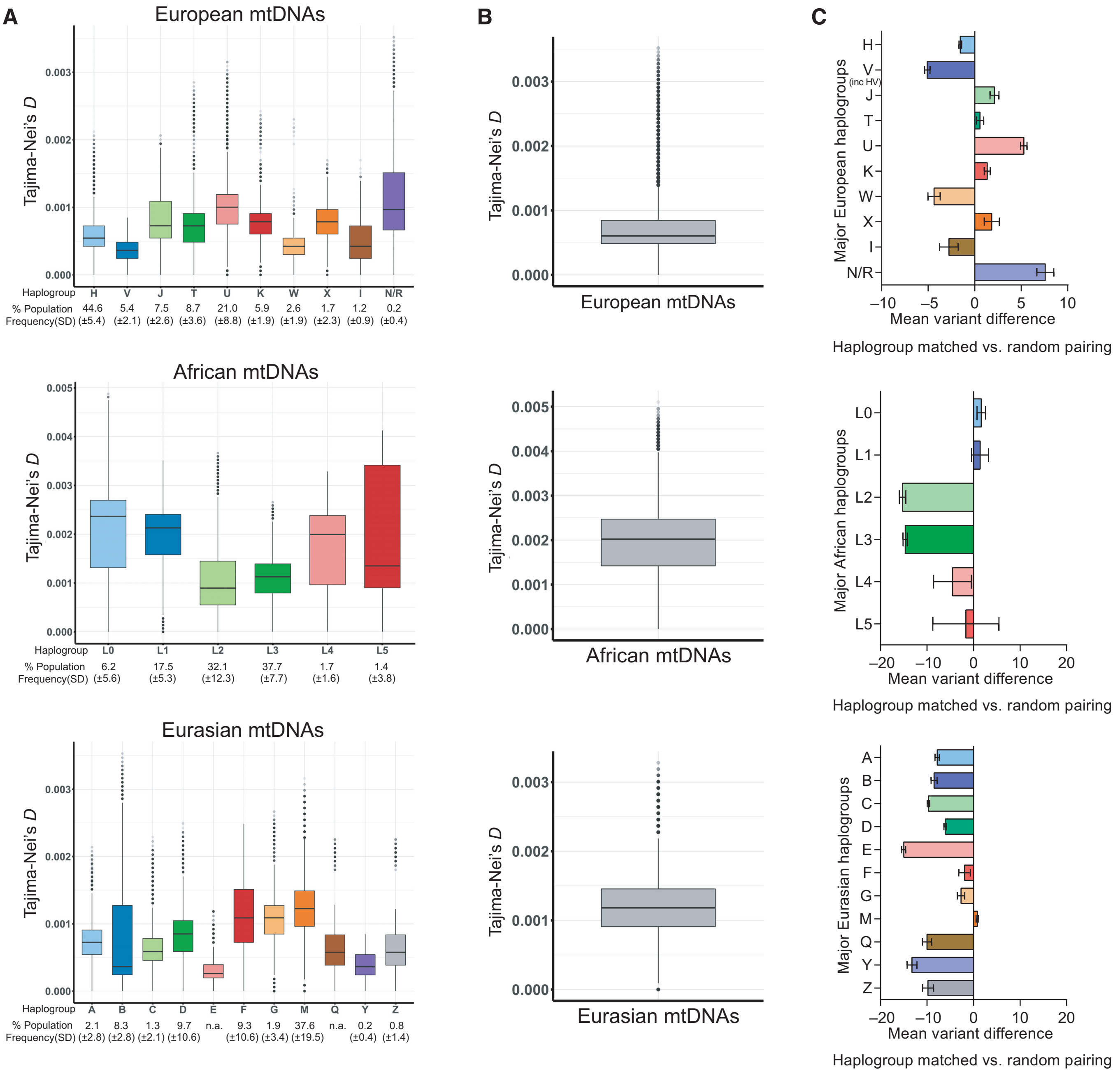

Figure 1. MtDNA sequence divergence within European, African, and Eurasian mtDNAs.

A Boxplots of the estimated pairwise mtDNA sequence divergence for the major European, African, and Eurasian haplogroups (i.e., haplogroup-matched mtDNAs, Dataset EV3A–C). The intra-haplogroup mtDNA sequence divergence of each population differs significantly (one-way ANOVA in each population $P < 2.2 \times 10^{-16}$).

B Boxplots of estimated pairwise mtDNA sequence divergence when two random sequences are selected within each population (i.e., unmatched mtDNAs, Dataset EV3A–C).

C Bar charts of the change in mean variant differences when mtDNA pairs are haplogroup matched (as in A) compared when they are randomly selected from the combined major European, African, and Eurasian haplogroups (as in B).

Data information: Boxplots show median, 25th and 75th percentile, with whiskers indicating 95th upper/lower interquartile range. Dots indicate outliers. Bar charts show mean, and standard deviation. Estimates (A and B) and counts (C) are based on 7,655 European, 3,688 African and 6,857 Eurasian mtDNA sequences. Population groups were defined by mtDNA haplogroup. Tajima-Nei's genetic distance model (Tajima-Nei's *D*) was used to derive sequence divergence, where Tajima-Nei's *D* 0.00006 = 1 variant difference.

© 2023 The Authors

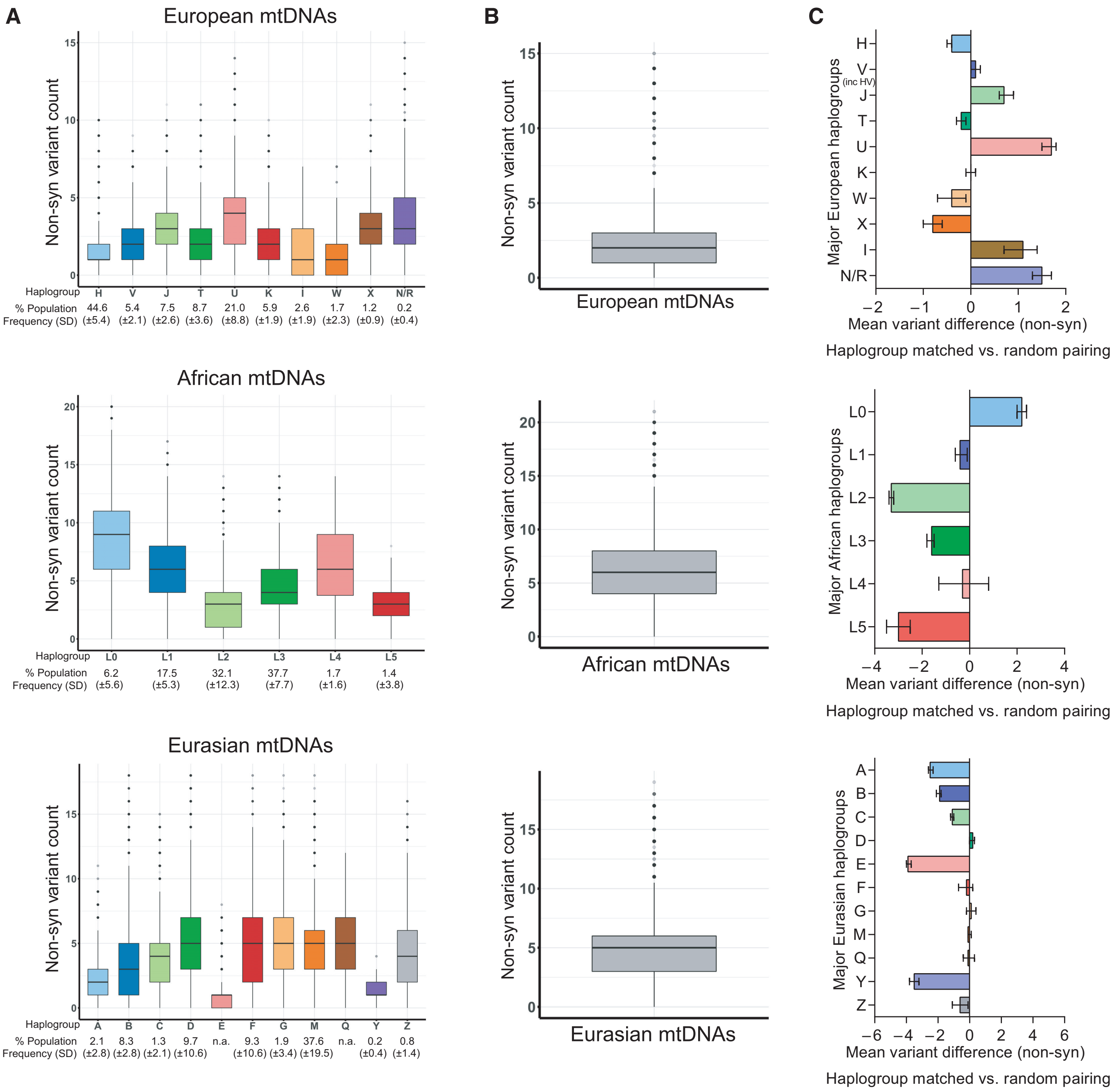

Figure 2. Non-synonymous variant differences divergence within European, African and Eurasian populations' mtDNAs.

A Boxplots showing the non-synonymous variant divergence within the major European, African, and Eurasian haplogroups (i.e., haplogroup matched mtDNAs, Dataset EV3A–C). The intra-haplogroup mtDNA sequence divergence of each population differs significantly (one-way ANOVA in each population $P < 2.2 \times 10^{-16}$).

B Boxplots showing the mean non-synonymous variant differences when two random sequences are selected within each population (i.e., unmatched mtDNAs, Dataset EV3A–C).

C Bar charts showing the mean non-synonymous variant differences when mtDNA pairs are haplogroup matched (as in A) compared when they are randomly selected from the combined major European, African, and Eurasian haplogroups (as in B).

Data information: Boxplots show median, 25th and 75th percentile, with whiskers indicating 95th upper/lower interquartile range. Dots indicate outliers. Bar charts show mean and standard deviation. Estimates (A and B) and counts (C) are based on 7,655 European, 3,688 African and 6,857 Eurasian mtDNA sequences. Population groups were defined by mtDNA haplogroup. Nei-Gojobori model was used to investigate non-synonymous variant differences.

 © 2023 The Authors

Table 2. Comparative non-synonymous variant differences between unmatched and haplogroup matched sequence pairs.

	Intra-group diversity						
	Estimated % population frequency (SD)	No. of sequences for divergence estimate	% of Dataset	Mean non-syn variant difference (95% CI)	Increased (▲) or Decreased (▼) compared to total dataset	Haplogroup versus total dataset *P*	Maximum non-syn variant differences
**A**							
Major European Haplogroups							
Total European Dataset	–	7655	–	2.1 (2.1–2.1)	–	–	15
H	44.6 (± 5.4)	3113	40.7%	1.7 (1.6–1.7)	▼	1.4E-05	10
V (inc HV)	5.4 (± 2.1)	420	5.5%	2.2 (2.1–2.3)	▲	5.6E-20	9
J	7.5 (± 2.6)	562	7.3%	2.8 (2.7–3.0)	▲	1.2E-20	11
T	8.7 (± 3.6)	675	8.8%	1.9 (1.8–2.0)	▼	4.3E-02	11
U	21.0 (± 8.8)	1285	16.8%	3.8 (3.6–3.9)	▲	9.1E-117	14
K	5.9 (± 1.9)	653	8.5%	2.1 (2.0–2.2)	–	n.s.	10
W	2.6 (± 1.9)	135	1.8%	1.7 (1.4–2.0)	▼	6.0E-02	7
X	1.7 (± 2.3)	142	1.9%	1.3 (1.1–1.5)	▼	1.8E-10	7
I	1.2 (± 0.9)	115	1.5%	3.2 (2.8–3.5)	▲	1.3E-08	11
N/R	0.2 (± 0.4)	555	7.3%	3.6 (3.4–3.8)	▲	2.4E-33	15
Major African Haplogroups							
Total African Dataset	–	3688	–	6.1 (6.0–6.2)	–	–	21
L0	6.2 (± 5.6)	983	26.7%	8.3 (8.1–8.5)	▲	2.5E-59	20
L1	17.5 (± 5.3)	704	19.1%	5.7 (5.5–6.0)	▼	2.1E-02	17
L2	32.1 (± 12.3)	835	22.6%	2.8 (2.7–2.9)	▼	3.3E-237	14
L3	37.7 (± 7.7)	1090	29.6%	4.5 (4.3–4.6)	▼	2.4E-65	14
L4	1.7 (± 1.6)	42	1.1%	5.8 (4.8–6.9)	–	n.s.	14
L5	1.4 (± 3.8)	34	0.9%	3.1 (2.6–3.6)	▼	2.0E-12	8
Major Eurasian Haplogroups							
Total Eurasian Dataset	–	6857	–	4.9 (4.9–5.0)	–	–	19
A	2.1 (± 2.8)	417	6.1%	2.4 (2.3–2.6)	▼	6.1E-122	11
B	8.3 (± 2.8)	994	14.5%	3.0 (2.8–3.1)	▼	1.4E-83	18
C	1.3 (± 2.1)	917	13.4%	3.8 (3.7–3.9)	▼	3.9E-34	15
D	9.7 (± 10.6)	1541	22.5%	5.1 (4.9–5.2)	–	n.s.	18
E	n.a.	234	3.4%	1.0 (0.9–1.2)	▼	1.6E-152	8
F	9.3 (± 10.6)	148	2.2%	4.7 (4.2–5.1)	–	n.s.	18
G	1.9 (± 3.4)	239	3.5%	5.0 (4.7–5.3)	–	n.s.	18
M	37.6 (± 19.5)	2061	30.1%	4.8 (4.8–5.0)	–	n.s.	18
Q	n.a.	156	2.3%	4.8 (4.5–5.2)	–	n.s.	12
Y	0.2 (± 0.4)	33	0.5%	1.4 (1.1–1.7)	▼	2.0E-20	4
Z	0.8 (± 1.4)	117	1.7%	4.3 (3.8–4.8)	–	n.s.	16
**B**							
African, European and Eurasian mtDNAs	–	18,200	–	3.9 (2.7–5.0)	–	–	20

© 2023 The Authors

Table 2 (continued)

	Intra-group diversity						
	Estimated % population frequency (SD)	No. of sequences for divergence estimate	% of Dataset	Mean non-syn variant difference (95% CI)	Increased (▲) or Decreased (▼) compared to total dataset	Haplogroup versus total dataset *P*	Maximum non-syn variant differences
European and African mtDNAs	-	11,343	63.3%	6.4 (6.4–6.5)	▲	1.2E-300	20
European and Eurasian mtDNAs	–	14,512	79.7%	4.6 (4.5–4.6)	▲	8.3E-28	19
African and Eurasian mtDNAs	–	10,454	57.4%	4.6 (4.5–4.6)	▲	9.1E-27	19

(A) Mean number of non-synonymous variant differences (with 95% CI) between randomly selected unmatched European, African, and Eurasian mtDNA pairs (greyed) and when sequence pairs are selected from within mtDNA (Dataset EV3A–C). Arrows indicate either an intra-haplogroup increase or decrease (green box) in non-synonymous variant differences relative to unmatched mtDNAs and P is the comparison of intra-haplogroup matched versus unmatched by Mann–Whitney *U*, dashes indicate no significant change. Shown is the number of sequences (and percentage frequency) used to make the divergence estimates and the maximum number variant differences observed between randomly paired sequences in each haplogroup. Population frequency is taken from phase 31,000 Genomes data is included (Dataset EV1A and B, where n.a. is not available, European = 503, African = 660 and Eurasian = 993 mtDNAs). (B) Mean number of non-synonymous variant differences (with 95% CI) between randomly selected unmatched mtDNA from all datasets (African, European, and Eurasian combined) and paired permutations of population group (e.g., African and European mtDNAs, Dataset EV3D).

Is mtDNA sequence divergence reduced by matching at the mtDNA subclade level?

The 30 major mtDNA haplogroups can be further classified into 'subclades', defined by further mtDNA variants (van Oven & Kayser, 2009). For example, haplogroup H can be divided into 106 subclades, denoted H1-H106 (van Oven & Kayser, 2009). Albeit perhaps unrealistic in practice, we asked whether matching donor/patient pairs according to mtDNA subclades would result in a greater reduction in sequence divergence compared with matching at the major haplogroup level. Although the level of mtDNA sequence divergence within subclades is reduced compared with major haplogroups (Appendix Fig S6A and B and Table S1), there remains considerable sequence variation within many subclades. Notably, 48% of European subclades (n = 50) show a significantly higher average variant differences when compared to randomly selected sequence pairs from the European population ($P < 0.05$, Appendix Table S1). Conversely, subclade matching would significantly reduce the average variant differences in the vast majority of African (94%, 18 out of 19) and Eurasian (82%, 46 of 56) subclades. However, the low frequency of most subclades (< 1%, 62 out of 125, Appendix Table S1) would render matching donor/patient pairs at the subclade level impractical.

Effect of matching donor/recipient pairs across mtDNA ancestries

As discussed above, the predominance of European mtDNA lineages among egg donors in the UK (HFEA, 2023) reduces the probability of finding a haplogroup-matched egg donor for patients with non-European maternal ancestry. We, therefore, determined the effect on mtDNA sequence divergence of using eggs from European haplogroup donors to treat women belonging to Eurasian or African haplogroups. By comparing with randomly sampled intra-population pairings, we find that the mean variant difference would be either reduced or unchanged by using eggs donated by European haplogroup donor to treat women belonging to African or Eurasian haplogroups respectively (Fig 3A). Conversely, the use of donor eggs with African or Eurasian haplogroups to treat European haplogroup patients would result in an increase in the mean mtDNA variant difference between patient/donor pairs (Fig 3A). These findings are consistent with previous studies showing that, although they share a subset of variants (Tishkoff & Williams, 2002; Tishkoff & Verrelli, 2003; Garrigan *et al*, 2007; Campbell & Tishkoff, 2008), mtDNA diversity among African haplogroups is increased compared with non-African haplogroups (Ingman *et al*, 2000; Tishkoff & Williams, 2002; Tishkoff & Verrelli, 2003; Garrigan *et al*, 2007). We conclude that pairing patients with African or Eurasian maternal ancestry with egg donors from across the European haplogroups would not increase the mean variant difference compared with randomly sampled mtDNA from the same phylogeographical region.

The above analysis relates to randomly samples pairs of mtDNA across all haplogroups and does not take account of variability in haplogroup frequencies between different populations (Fig 1B). We, therefore, investigated the effect of inter-population mixes on variant differences between all common major haplogroups. The mean variant difference between randomly sampled pairs across haplogroups varied from 10 (between haplogroups H/L3) to 50 (between haplogroups A/L1; Fig 3B). In many cases, the mean variant difference is not reduced by matching non-European haplogroups with haplogroup H (Fig 3B). Taken together these findings indicate that the prevalence of European mtDNA lineages among egg donors in Europe, should not be a barrier to MRT treatment for women belonging to African or Eurasian haplogroups, based on concerns about mtDNA sequence divergence between them.

Discussion

The development of MRT offers women for whom PGT is unsuitable, the potential to reduce the risk of transmitting serious mtDNA

 © 2023 The Authors

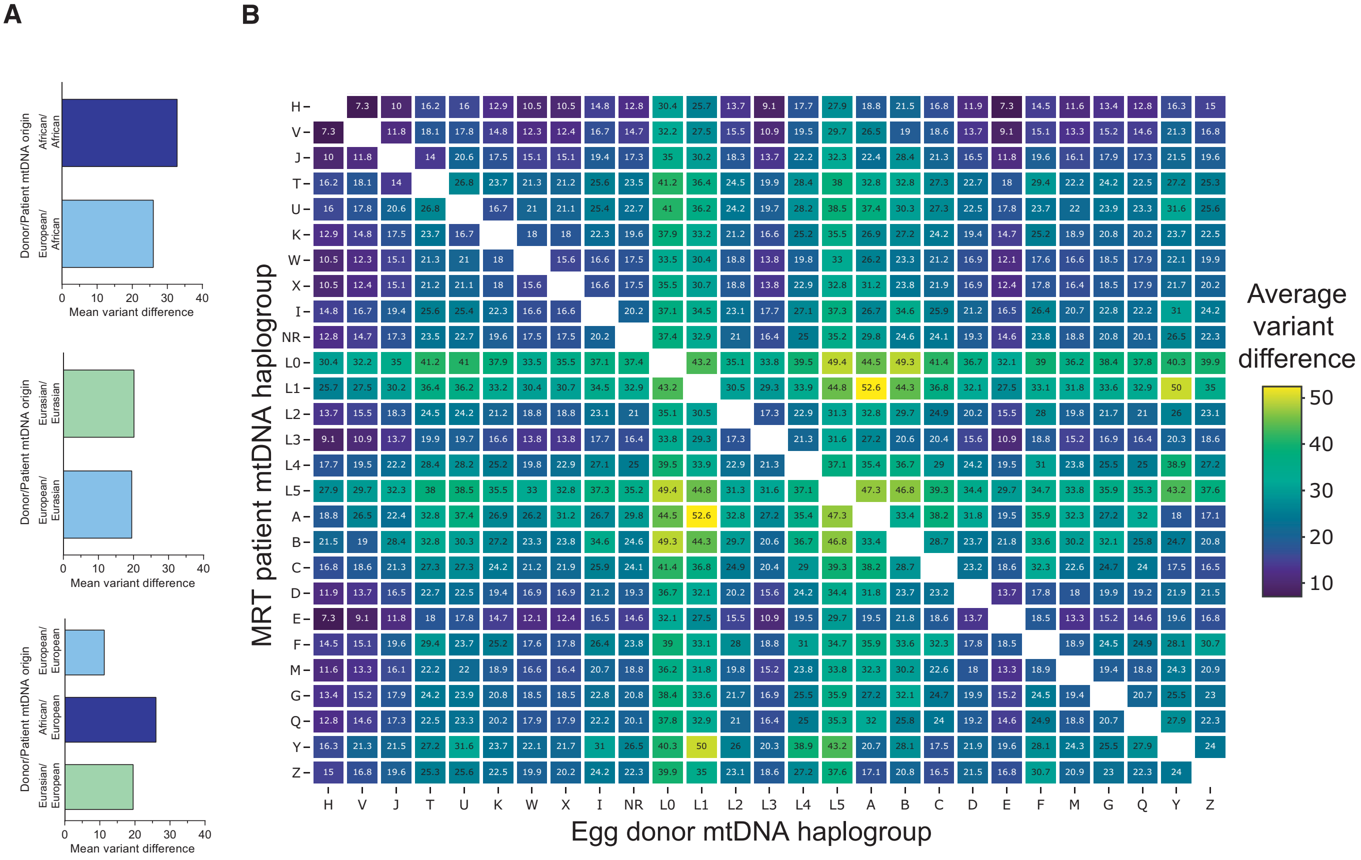

Figure 3. Non-synonymous variant differences divergence within European, African and Eurasian populations' mtDNAs.

A Graphs showing the average total mtDNA variation observed when donor/recipient mtDNA pairs are selected from across two populations (Dataset EV3E). The top and middle graphs show average differences when a patient belonging to an African or Eurasian-origin haplogroup respectively is matched with an egg donor from a European haplogroup compared with a population-matched egg donor. The lower graphs show the average differences when a patient belonging to a European haplogroup is matched with an egg donor belonging to an African, Eurasian, or European haplogroup.

B Heatmap showing the average variant differences between pairs of mtDNAs selected from all common haplogroups in each population (Dataset EV4).

disease to their children. However, the supply of donated eggs is already a limiting factor, and our findings demonstrate that this problem would be greatly exacerbated by mtDNA haplogroup matching between egg donors and women undergoing MRT treatment. We find that the impact of haplogroup matching would be particularly severe for women belonging to rare haplogroups, who may have to wait several years for a haplogroup matched donor. Strikingly, comparison of haplogroup matched and randomly selected mtDNA pairs reveals comparable levels of sequence divergence for many haplogroups.

To interrogate the potential functional relevance, we asked whether haplogroup matching might specifically reduce the levels of non-synonymous sequence variants. Our findings indicate that non-synonymous variant differences between haplogroup-matched pairs are comparable with randomly samples pairs from combined haplogroups within each population. Similarly, mtDNA sequence divergence between patient/donor pairs would not be eliminated by increasing the resolution to match at the subclade level. Together these findings indicate that haplogroup matching at the major haplogroup, or subclade level would severely constrain the delivery of an MRT programme, without necessarily reducing either total or non-synonymous mtDNA sequence divergence between egg donors and women undergoing MRT.

In broad terms, an individual's mtDNA haplogroup is associated with the continental ancestry of their maternal lineage (Emery *et al*, 2015). Our findings indicate that women having MRT treatment in Northern Europe may wait up to 9 years for eggs from a donor belonging to the major haplogroup L, which accounts for > 92% of African populations (Dataset EV1A). The problem of finding haplogroup matched egg donors for women from diverse phylogeographical regions may in theory be alleviated by importing vitrified eggs from egg banks around the world. Whilst egg vitrification is a successful procedure (Rafael *et al*, 2022), the use of vitrified donor eggs for MRT is associated with increased carryover of mitochondria (Hyslop *et al*, 2016), which can result in elevated heteroplasmy for maternal mtDNA in babies born after MRT (Costa-Borges *et al*, 2023). Thus, although, future advances in the use of vitrified donor eggs for MRT may enable import from international egg banks, current clinical practice relies largely on local egg donors, which are overwhelming of European maternal ancestry (HFEA, 2023). Encouragingly, analysis of all possible combinations of haplogroups indicates that matching Eurasian or African

© 2023 The Authors

haplogroups with any European haplogroup, notably the most prevalent (major haplogroup H), results in mean variant differences that are in the lower range (10–20 vs. 50 for some intra-population mixes). Thus, the use of donated eggs from European haplogroups in MRT treatment for women with African and Eurasian maternal ancestry is unlikely to increase the number of mtDNA sequence variants compared with donated eggs from the same phylogeographical region.

Whether differences in continental ancestry between donor/recipient pairs might have consequences beyond sequence divergence is unclear. It has been reported that children born to couples from different continental ancestries acquire mtDNA variant signatures that correspond to the nuclear genome's 'native' mtDNA (Wei *et al*, 2019). This raises the possibility that the nuclear genome somehow shapes the mitochondrial genome. Whilst it remains to be established whether this occurs after MRT, *de novo* variants have not been detected in embryonic stem (ES) cell lines derived from MRT embryos, irrespective of whether the donor/recipient pairs belong to the same, or different ancestries (Hyslop *et al*, 2016; Kang *et al*, 2016; Yamada *et al*, 2016). Such ES cell lines may provide a useful experimental system for further investigating the 'nuclear entrained' acquisition of mtDNA variants.

Concerns regarding the biological consequences of creating new combinations of mtDNA and nuclear DNA during MRT are largely based on observations from studies on conplastic flies (Clancy, 2008; Zhang *et al*, 2017) and mice (Latorre-Pellicer *et al*, 2016). Reports form these studies indicate that backcrossing the nuclear genome of one inbred strain onto the cytoplasm of another generally reduces fitness, resulting in ovarian failure, embryonic lethality (Zhang *et al*, 2017), reduced longevity (Clancy, 2008; Latorre-Pellicer *et al*, 2016), and altered metabolic and mitochondrial function (Latorre-Pellicer *et al*, 2016). Based on these findings, it was suggested that similar effects may arise from MRT-induced disruption of the interplay between the egg's nuclear and mitochondrial genomes (Burgstaller *et al*, 2015; Morrow *et al*, 2015; Latorre-Pellicer *et al*, 2016; Royrvik *et al*, 2016). However, female meiosis involves loss of 75% of the maternal genome in the polar bodies (Herbert *et al*, 2015) and there is no known mechanism to preferentially retain maternally inherited nuclear genes 'native' to the oocyte mtDNA. In this sense, that scope for interplay based on coevolution of nuclear and mitochondrial genomes (Clancy, 2008; Latorre-Pellicer *et al*, 2016; Zhang *et al*, 2017) may not extend beyond one generation in outbred species. Notably, in contrast to laboratory flies and mice, sexual reproduction in humans may involve the creation of combinations of nuclear DNA and mtDNA from diverse ancestries. Based on population studies, this is not a barrier to human health (Eyre-Walker, 2017; Rishishwar & Jordan, 2017), indeed, it has been reported that children born from mixed-ancestry parents typically demonstrate increased fitness (Campbell *et al*, 2007; Lewis, 2010).

In summary, our findings indicate that intra-haplogroup sequence variation limits the extent to which haplogroup matching can reduce sequence divergence between egg donors and women undergoing MRT. Considering the general shortage of egg donors (Platts *et al*, 2021), haplogroup matching could impose a delay of several years in accessing MRT treatment. We find that women belonging to rare haplogroups would be worst affected but would stand to gain the least owing to the generally high levels of sequence variation within rare haplogroups. Given the likely negative impact on the availability of donor eggs, we propose that haplogroup matching is not warranted for the purpose of reducing the number of mtDNA sequence variants between donor/recipient pairs. Our analysis does not take account of the potential impact of specific variants. Although there are major gaps in knowledge of the functional relevance of non-pathogenic variants either individually or in combination (Chinnery & Gomez-Duran, 2018; McCormick *et al*, 2020), haplogroup-defining variants are predominantly synonymous (Elson *et al*, 2004; Wei *et al*, 2017), limiting the scope for functional implications. However, it should be noted that non-haplogroup defining variants may influence the outcome of MRT, especially in relation the fate of maternal mtDNA co-transplanted with the nuclear genome (Hudson *et al*, 2019). Thus, it will be important to monitor the impact of mtDNA sequence variants, including haplogroup combinations on MRT outcomes over the longer term. Finally, since MRT involves swapping the entire contents of the egg cytoplasm, detrimental or indeed beneficial effects (Costa-Borges *et al*, 2023), may be unrelated to the mitochondria.

Materials and Methods

MtDNA haplogroup frequencies

To demonstrate mtDNA haplogroup and subclade diversity both 'globally' and 'locally', we used two independent datasets. Global mtDNA haplogroup frequencies were estimated using phase3 1,000 Genomes data (https://www.internationalgenome.org/home), a publicly available repository of genome variant data arranged by population, region or country. MtDNA variant data (ALL.chrMT.phase3*genotypes.vcf*) for 2,504 individuals from 28 populations were downloaded and mtDNA haplogroups were determined using HaploGrep2 (Kloss-Brandstatter *et al*, 2011; utilising PhyloTree v.17). Summarised Global haplogroup data, stratified by country, is available in Dataset EV1A and as individuals in Dataset EV1B. We used the European population as an exemplar to investigate the potential impact of 'local' haplogroup-matching and sub-clade on donor availability in the UK. European mtDNA haplogroup frequencies were downloaded from Eupedia (https://www.eupedia.com/europe/european_mtdna_haplogroups_frequency.shtml), a publicly available repository of published mtDNA haplogroup data arranged by population and country. Summarised European haplogroup data, stratified by country, is available in Dataset EV1A and as individuals in Dataset EV1B.

MtDNA haplogroup sequence divergence

We used Using Tajima-Nei's (Tajima & Nei, 1983) genetic distance model and Nei-Gojobori's (Nei & Gojobori, 1986) method to estimate total mtDNA and non-synonymous sequence diversity between all pairs of mtDNAs in three population groups. Whole Human mtDNA genome data, ~30,000 sequences, were downloaded from the National Centre for Biotechnology Information Nucleotide database (www.ncbi.nlm.nih.gov), using the keyword phrase 'Homo [Organism] AND gene_in_mitochondrion[PROP] AND

 © 2023 The Authors

14000:19000[SLEN] NOT pseudogene[All Fields]'. Sequences with known pathogenic mtDNA variants (available at www.mitomap.org) and non-homo sapiens sequences were removed. Duplicated sequences (same reference, but different GeneInfo identification number) were removed and only a single mtDNA from familial uploads was included. The trimmed sequence dataset was aligned using MUSCLE (Edgar, 2004), analysed using HaploGrep2 (Kloss-Brandstatter *et al*, 2011) to assign major mtDNA haplogroups (i.e. H) and haplogroup subclades (i.e. H1) (utilising PhyloTree v.17) and subsequently filtered to match the major European haplogroups (H, V, J, T, U, K, W, X, I, R and N, leaving a final sequence dataset of 7,655 samples, Dataset EV3A), African haplogroups (L0, L1, L2, L3, L4 and L5, leaving a final sequence dataset of 3,688 samples, Dataset EV3B) and Eurasian haplogroups (A, B, C, D, E, F, G, M, Q, Y and Z, leaving a final sequence dataset of 6,857 samples, Dataset EV3C) as per www.mitomap.org.

mtDNA sequence divergences were determined by pairwise comparison of whole mitochondrial genomes using MEGA (v.7.0; Kumar *et al*, 2008) and the Tajima-Nei model (Nei & Gojobori, 1986). This approximates the divergence for a randomly chosen pair of individuals with that haplogroup or haplogroup subclade (Nei & Gojobori, 1986). Non-synonymous variant counts were calculated using the Nei-Gojobori model (Nei & Gojobori, 1986). Summary data within haplogroups and haplogroup subclades (i.e. haplogroup-matched, > 10 sequences per group or MAF > 0.1%) and in each population group (i.e. all unmatched sequences), including mean, standard deviation, variance, median, percentiles (25th and 75th), minimum & maximum distance, and range of distances were generated and plotted in R (v.3.4.3; Ginestet, 2011; R Core Team, 2013; Dataset EV3A–D). Variant differences (total and non-synonymous) between haplogroups and haplogroup subclades were compared to each respective unmatched group by Mann–Whitney *U* and adjusted for multiple significant testing by Bonferroni correction. All plots were generated in R (v.3.4.3; Ginestet, 2011; R Core Team, 2013) using the packages ggplot2 and heatmaply.

Data availability

This study includes no data deposited in external repositories.

Expanded View for this article is available online.

Acknowledgements
MH and GH are supported by the Wellcome Centre for Mitochondrial Research (203105/Z/16/Z). LH (ICA Clinical Lectureship, ICA-CL-2017-03-010) was funded by Health Education England (HEE)/National Institute for Health Research (NIHR).

Author contributions
Yuko Takeda: Conceptualization; formal analysis; visualization; methodology; writing – original draft; writing – review and editing. **Louise Hyslop:** Data curation; formal analysis; methodology; writing – original draft; writing – review and editing. **Meenakshi Choudhary:** Data curation; formal analysis; methodology; writing – original draft; writing – review and editing. **Fiona Robertson:** Software; formal analysis; visualization; methodology; writing – original draft; writing – review and editing. **Angela Pyle:** Data curation; supervision; investigation; methodology; writing – original draft; writing – review and editing. **Ian Wilson:** Formal analysis; investigation; methodology; writing – original draft; writing – review and editing. **Mauro Santibanez-Koref:** Formal analysis; investigation; visualization; methodology; writing – original draft; writing – review and editing. **Douglass Turnbull:** Investigation; writing – original draft; writing – review and editing. **Mary Herbert:** Conceptualization; resources; formal analysis; methodology; writing – original draft; project administration; writing – review and editing. **Gavin Hudson:** Conceptualization; formal analysis; supervision; investigation; visualization; methodology; writing – original draft; project administration; writing – review and editing.

Disclosure and competing interests statement
The authors declare that they have no conflict of interest.

References

Burgstaller JP, Johnston IG, Poulton J (2015) Mitochondrial DNA disease and developmental implications for reproductive strategies. *Mol Hum Reprod* 21: 11 – 22

Cai N, Gomez-Duran A, Yonova-Doing E, Kundu K, Burgess AI, Golder ZJ, Calabrese C, Bonder MJ, Camacho M, Lawson RA *et al* (2021) Mitochondrial DNA variants modulate N-formylmethionine, proteostasis and risk of late-onset human diseases. *Nat Med* 27: 1564 – 1575

Campbell MC, Tishkoff SA (2008) African genetic diversity: implications for human demographic history, modern human origins, and complex disease mapping. *Annu Rev Genomics Hum Genet* 9: 403 – 433

Campbell H, Carothers AD, Rudan I, Hayward C, Biloglav Z, Barac L, Pericic M, Janicijevic B, Smolej-Narancic N, Polasek O *et al* (2007) Effects of genome-wide heterozygosity on a range of biomedically relevant human quantitative traits. *Hum Mol Genet* 16: 233 – 241

Chinnery PF, Gomez-Duran A (2018) Oldies but goldies mtDNA population variants and neurodegenerative diseases. *Front Neurosci* 12: 682

Chinnery PF, Hudson G (2013) Mitochondrial genetics. *Br Med Bull* 106: 135 – 159

Clancy DJ (2008) Variation in mitochondrial genotype has substantial lifespan effects which may be modulated by nuclear background. *Aging Cell* 7: 795 – 804

Costa-Borges N, Nikitos E, Spath K, Miguel-Escalada I, Ma H, Rink K, Coudereau C, Darby H, Koski A, Van Dyken C *et al* (2023) First pilot study of maternal spindle transfer for the treatment of repeated in vitro fertilization failures in couples with idiopathic infertility. *Fertil Steril* 119: 964 – 973

Edgar RC (2004) MUSCLE: a multiple sequence alignment method with reduced time and space complexity. *BMC Bioinformatics* 5: 113

Elson JL, Turnbull DM, Howell N (2004) Comparative genomics and the evolution of human mitochondrial DNA: assessing the effects of selection. *Am J Hum Genet* 74: 229 – 238

Emery LS, Magnaye KM, Bigham AW, Akey JM, Bamshad MJ (2015) Estimates of continental ancestry vary widely among individuals with the same mtDNA haplogroup. *Am J Hum Genet* 96: 183 – 193

Eyre-Walker A (2017) Mitochondrial replacement therapy: are mito-nuclear interactions likely to be a problem? *Genetics* 205: 1365 – 1372

Garrigan D, Kingan SB, Pilkington MM, Wilder JA, Cox MP, Soodyall H, Strassmann B, Destro-Bisol G, de Knijff P, Novelletto A *et al* (2007) Inferring human population sizes, divergence times and rates of gene flow from mitochondrial, X and Y chromosome resequencing data. *Genetics* 177: 2195 – 2207

© 2023 The Authors

Ginestet C (2011) ggplot2: elegant graphics for data analysis. *J R Stat Soc Stat* 174: 245 – 246

Gomez-Duran A, Pacheu-Grau D, Martinez-Romero I, Lopez-Gallardo E, Lopez-Perez MJ, Montoya J, Ruiz-Pesini E (2012) Oxidative phosphorylation differences between mitochondrial DNA haplogroups modify the risk of Leber's hereditary optic neuropathy. *Biochim Biophys Acta* 1822: 1216 – 1222

Gorman GS, Chinnery PF, DiMauro S, Hirano M, Koga Y, McFarland R, Suomalainen A, Thorburn DR, Zeviani M, Turnbull DM (2016) Mitochondrial diseases. *Nature Reviews Disease Primers* 2: 16080

Greenfield A (2016) Scientific review of the safety and efficacy of methods to avoid mitochondrial disease: 2016 update, in: Authority H.F.a.E. (Ed.). www.hfea.gov.uk

Greenfield A, Braude P, Flinter F, Lovell-Badge R, Ogilvie C, Perry ACF (2017) Assisted reproductive technologies to prevent human mitochondrial disease transmission. *Nat Biotechnol* 35: 1059 – 1068

Herbert M, Turnbull D (2015) Mitochondrial replacement to prevent the transmission of mitochondrial DNA disease. *EMBO Rep* 16: 539 – 540

Herbert M, Kalleas D, Cooney D, Lamb M, Lister L (2015) Meiosis and maternal aging: insights from aneuploid oocytes and trisomy births. *Cold Spring Harb Perspect Biol* 7: a017970

Herbert M, Choudhary M, Zander-Fox D (2023) Assisted reproductive technologies at the nexus of fertility treatment and disease prevention. *Science* 380: 164 – 167

HFEA (2023) *Ethnic diversity in fertility treatment 2018*. London: The Human Fertilisation and Embryology Authority

Hudson G, Takeda Y, Herbert M (2019) Reversion after replacement of mitochondrial DNA. *Nature* 574: E8 – E11

Hyslop LA, Blakeley P, Craven L, Richardson J, Fogarty NM, Fragouli E, Lamb M, Wamaitha SE, Prathalingam N, Zhang Q *et al* (2016) Towards clinical application of pronuclear transfer to prevent mitochondrial DNA disease. *Nature* 534: 383 – 386

Ingman M, Kaessmann H, Paabo S, Gyllensten U (2000) Mitochondrial genome variation and the origin of modern humans. *Nature* 408: 708 – 713

Kang E, Wu J, Gutierrez NM, Koski A, Tippner-Hedges R, Agaronyan K, Platero-Luengo A, Martinez-Redondo P, Ma H, Lee Y *et al* (2016) Mitochondrial replacement in human oocytes carrying pathogenic mitochondrial DNA mutations. *Nature* 540: 270 – 275

Kloss-Brandstatter A, Pacher D, Schonherr S, Weissensteiner H, Binna R, Specht G, Kronenberg F (2011) HaploGrep: a fast and reliable algorithm for automatic classification of mitochondrial DNA haplogroups. *Hum Mutat* 32: 25 – 32

Kumar S, Nei M, Dudley J, Tamura K (2008) MEGA: a biologist-centric software for evolutionary analysis of DNA and protein sequences. *Brief Bioinform* 9: 299 – 306

Latorre-Pellicer A, Moreno-Loshuertos R, Lechuga-Vieco AV, Sanchez-Cabo F, Torroja C, Acin-Perez R, Calvo E, Aix E, Gonzalez-Guerra A, Logan A *et al* (2016) Mitochondrial and nuclear DNA matching shapes metabolism and healthy ageing. *Nature* 535: 561 – 565

Lee C, Mandoiu II, Nelson CE (2011) Inferring ethnicity from mitochondrial DNA sequence. *BMC Proc* 5: S11

Lewis MB (2010) Why are mixed-race people perceived as more attractive? *Perception* 39: 136 – 138

McCormick EM, Lott MT, Dulik MC, Shen L, Attimonelli M, Vitale O, Karaa A, Bai R, Pineda-Alvarez DE, Singh LN *et al* (2020) Specifications of the ACMG/AMP standards and guidelines for mitochondrial DNA variant interpretation. *Hum Mutat* 41: 2028 – 2057

Morrow EH, Reinhardt K, Wolff JN, Dowling DK (2015) Risks inherent to mitochondrial replacement. *EMBO Rep* 16: 541 – 544

Nei M, Gojobori T (1986) Simple methods for estimating the numbers of synonymous and nonsynonymous nucleotide substitutions. *Mol Biol Evol* 3: 418 – 426

van Oven M, Kayser M (2009) Updated comprehensive phylogenetic tree of global human mitochondrial DNA variation. *Hum Mutat* 30: E386 – E394

Pennings G, de Mouzon J, Shenfield F, Ferraretti AP, Mardesic T, Ruiz A, Goossens V (2014) Socio-demographic and fertility-related characteristics and motivations of oocyte donors in eleven European countries. *Hum Reprod* 29: 1076 – 1089

Platts S, Bracewell-Milnes T, Saso S, Jones B, Parikh R, Thum MY (2021) Investigating attitudes towards oocyte donation amongst potential donors and the general population: a systematic review. *Hum Fertil (Camb)* 24: 169 – 181

R Core Team (2013) *R: a language and environment for statistical computing*. Vienna: R Foundation for Statistical Computing

Rafael F, Robles GM, Navarro AT, Garrido N, Garcia-Velasco JA, Bosch E, Nunes SG, Soares SR, Santos-Ribeiro S (2022) Perinatal outcomes in children born after fresh or frozen embryo transfer using donated oocytes. *Hum Reprod* 37: 1642 – 1651

Ramos A, Santos C, Mateiu L, Gonzalez Mdel M, Alvarez L, Azevedo L, Amorim A, Aluja MP (2013) Frequency and pattern of heteroplasmy in the complete human mitochondrial genome. *PLoS One* 8: e74636

Reidla M, Kivisild T, Metspalu E, Kaldma K, Tambets K, Tolk HV, Parik J, Loogvali EL, Derenko M, Malyarchuk B *et al* (2003) Origin and diffusion of mtDNA haplogroup X. *Am J Hum Genet* 73: 1178 – 1190

Rishishwar L, Jordan IK (2017) Implications of human evolution and admixture for mitochondrial replacement therapy. *BMC Genomics* 18: 140

Royrvik EC, Burgstaller JP, Johnston IG (2016) mtDNA diversity in human populations highlights the merit of haplotype matching in gene therapies. *Mol Hum Reprod* 22: 809 – 817

Sachs P, Covington SN, Toll C, Richter KS, Purcell M, Chang FE (2010) Demographic variables related to successful anonymous oocyte donor recruitment. *Fertil Steril* 93: 311 – 313

Smeets HJM, Sallevelt SCEH, Dreesen JCFM, de Die-Smulders CEM, de Coo IFM (2015) Preventing the transmission of mitochondrial DNA disorders using prenatal or preimplantation genetic diagnosis. *Ann N Y Acad Sci* 1350: 29 – 36

Soares P, Ermini L, Thomson N, Mormina M, Rito T, Rohl A, Salas A, Oppenheimer S, Macaulay V, Richards MB (2009) Correcting for purifying selection: an improved human mitochondrial molecular clock. *Am J Hum Genet* 84: 740 – 759

Tajima F, Nei M (1983) Estimation of evolutionary distance between nucleotide-sequences. *Japan J Genet* 58: 684 – 685

Tishkoff SA, Verrelli BC (2003) Patterns of human genetic diversity: implications for human evolutionary history and disease. *Annu Rev Genomics Hum Genet* 4: 293 – 340

Tishkoff SA, Williams SM (2002) Genetic analysis of African populations: human evolution and complex disease. *Nat Rev Genet* 3: 611 – 621

Wei W, Gomez-Duran A, Hudson G, Chinnery PF (2017) Background sequence characteristics influence the occurrence and severity of disease-causing mtDNA mutations. *PLoS Genet* 13: e1007126

Wei W, Tuna S, Keogh MJ, Smith KR, Aitman TJ, Beales PL, Bennett DL, Gale DP, Bitner-Glindzicz MAK, Black GC *et al* (2019) Germline selection shapes human mitochondrial DNA diversity. *Science* 364: eaau6520

Yamada M, Emmanuele V, Sanchez-Quintero MJ, Sun B, Lallos G, Paull D, Zimmer M, Pagett S, Prosser RW, Sauer MV *et al* (2016) Genetic drift can

© 2023 The Authors

compromise mitochondrial replacement by nuclear transfer in human oocytes. *Cell Stem Cell* 18: 749 – 754
Zhang C, Montooth KL, Calvi BR (2017) Incompatibility between mitochondrial and nuclear genomes during oogenesis results in ovarian failure and embryonic lethality. *Development* 144: 2490 – 2503

License: This is an open access article under the terms of the Creative Commons Attribution License, which permits use, distribution and reproduction in any medium, provided the original work is properly cited.

Expanded View Figures

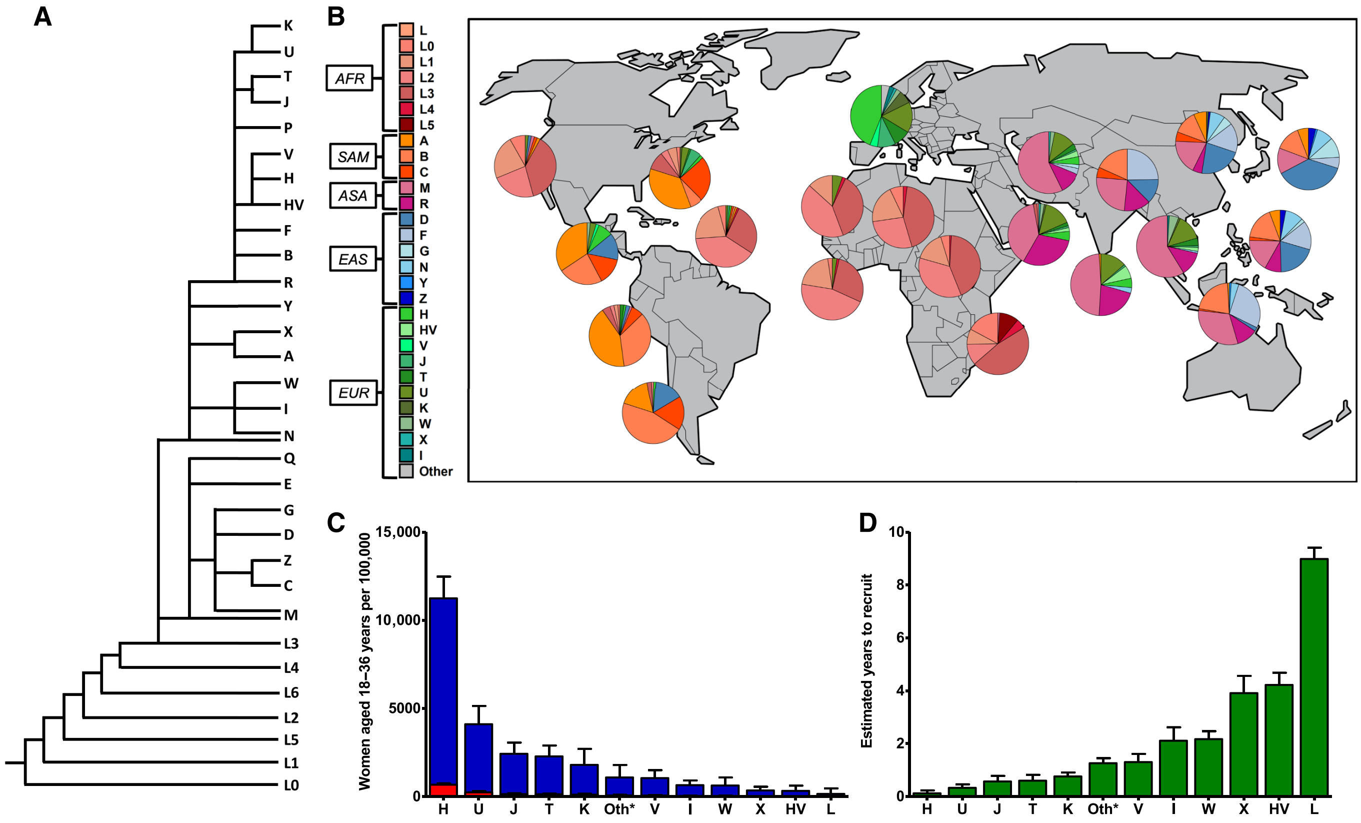

Figure EV1. Impact of haplogroup matching on the availability of egg donors for MRT.

A Simplified mtDNA haplogroup phylogeny showing the 31 'major' mtDNA haplogroups (phylotree.org).

B Graphical representation of global mtDNA haplogroup diversity, showing the relative estimated proportions of the most frequent mtDNA haplogroups by region (where available, AFR = Africa, SAM = South America, ASA = Southern Asia, EAS = East Asia and EUR = Europe, Dataset EV1A and B).

C Graph showing the estimated number of women per 100,000 that proceed to egg donation (red) in relation to those that are eligible to donate (blue) for each of the commonest mtDNA haplogroups identified across European populations (Based on 316 donors, Datasets EV1A and EV2A, and assuming the haplogroup of those who donate eggs is reflective of ancestry).

D Estimated years to recruit one volunteer per European mtDNA haplogroup based on the estimated donor availability and the estimated number of women who progress to egg donation (Datasets EV1A and EV2A).

Data information: Bar graphs show mean and standard deviation.

 © 2023 The Authors

Appendix Figures

Contents:

- **Appendix figure S1 | European mtDNA haplogroup distributions.**
- **Appendix figure S2 | Proportion of women per 100,000 per haplogroup.**
- **Appendix figure S3 | UK egg donors by reported ethnicity.**
- **Appendix figure S4 | mtDNA haplogroup diversity versus estimated population frequency.**
- **Appendix figure S5 | mtDNA haplogroup diversity versus estimated evolutionary age.**
- **Appendix figure S6 | Total mtDNA variant sequence divergence within European, African, and Eurasian mtDNA haplogroup subclades.**
- **Appendix table S1 | Mean mtDNA variant differences between unmatched and haplogroup matched sequence pairs.**

Appendix figure S1

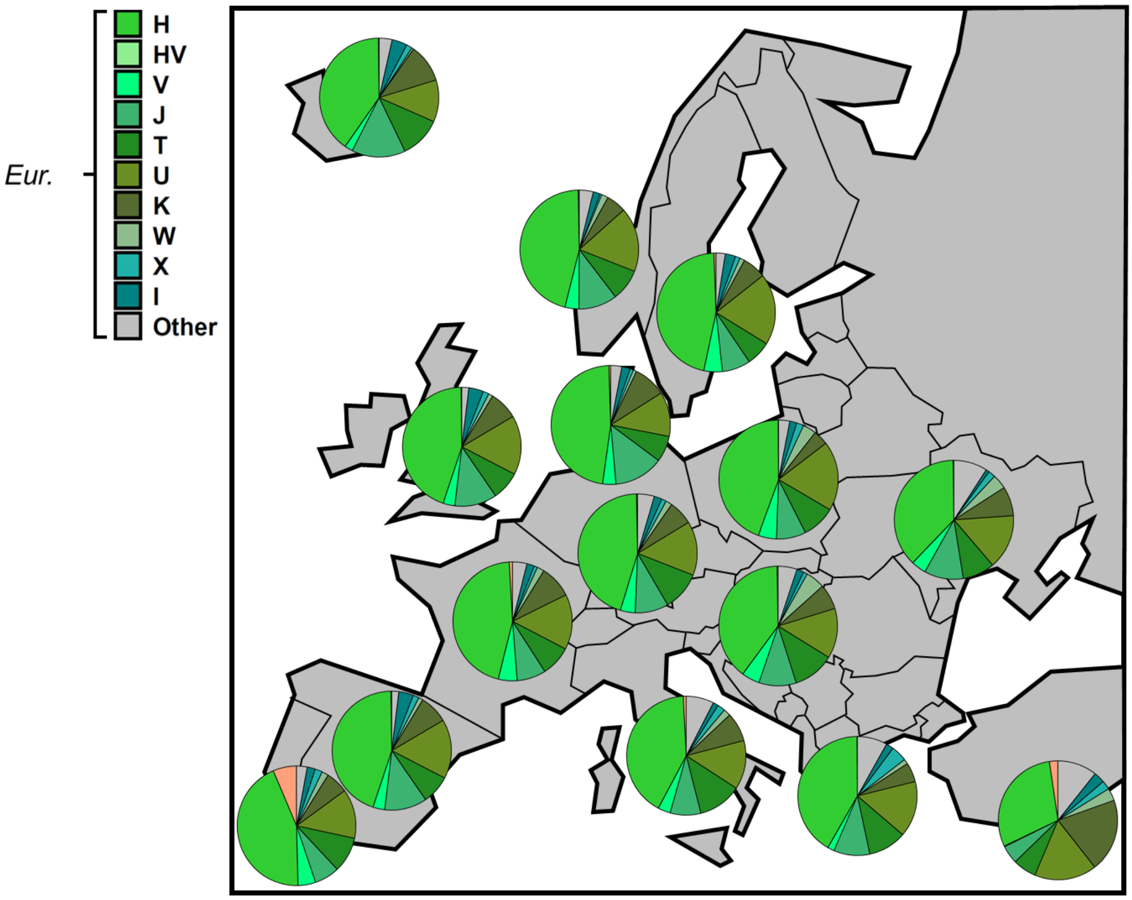

Appendix figure S1 | European mtDNA haplogroup distributions.
Graphical representation of the relative frequency of the 10 major European (Eur) mtDNA haplogroups, which are most likely to contribute to a local donor pool (haplogroups H, HV, V, J, T, U, K, I, W and X), per country (**Dataset EV1a**). In this instance, due to low proportional frequency, 'Other' includes both African and Asian haplogroups).

Appendix figure S2

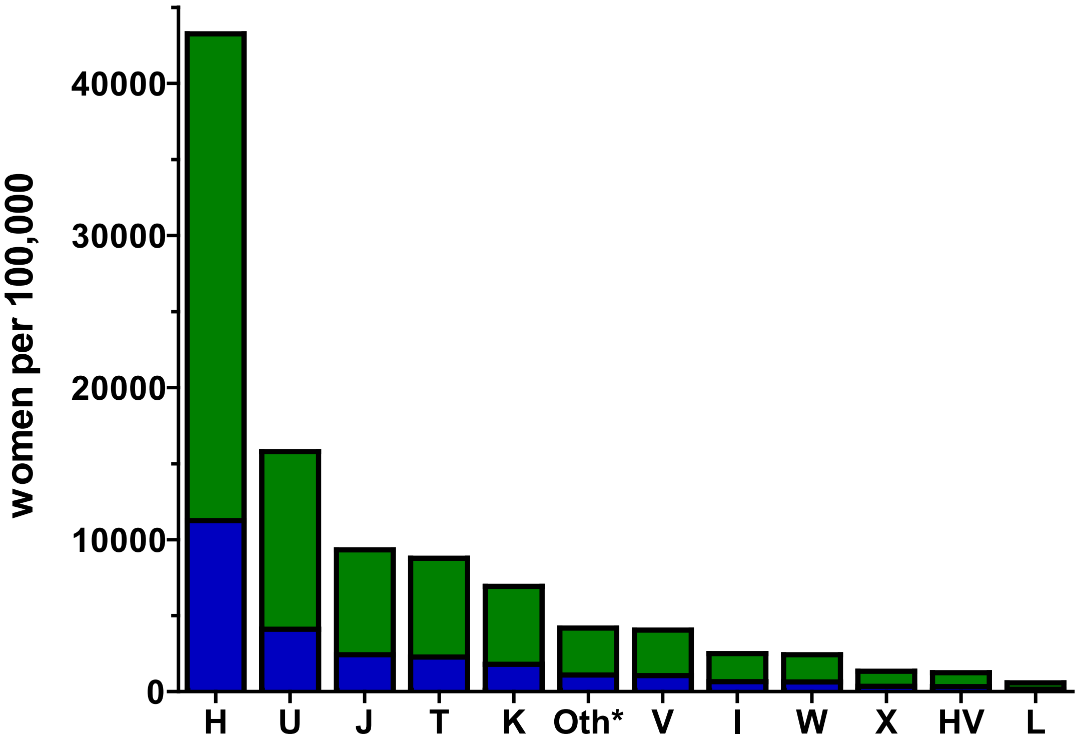

Appendix figure S2 | Proportion of women per 100,000 per haplogroup.
Graph showing the estimated proportion of women per 100,000 in each of the major mtDNA haplogroups in the European population (green) and the proportion of those who would be within the typical age range for egg donation (18-36 years, in blue) (**Dataset EV2a**).

Appendix figure S3

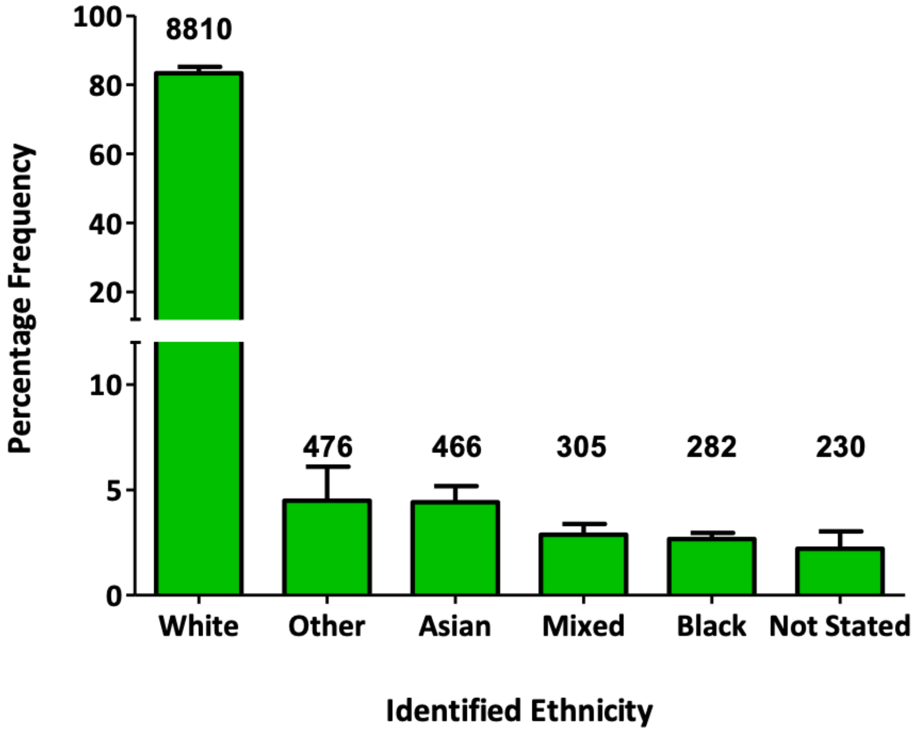

Appendix figure S3 | UK egg donors by reported ethnicity.
Bar graph showing the mean proportion and 95% CI (whiskers) of UK egg donor ethnicities ranked by frequency. The mean and confidence interval represent egg donations over a 5-year period (**Dataset EV2b**). The numbers indicate total number of egg donors in each group obtained from the HFEA report, “HFEA key donor information update 2017.”

Appendix figure S4

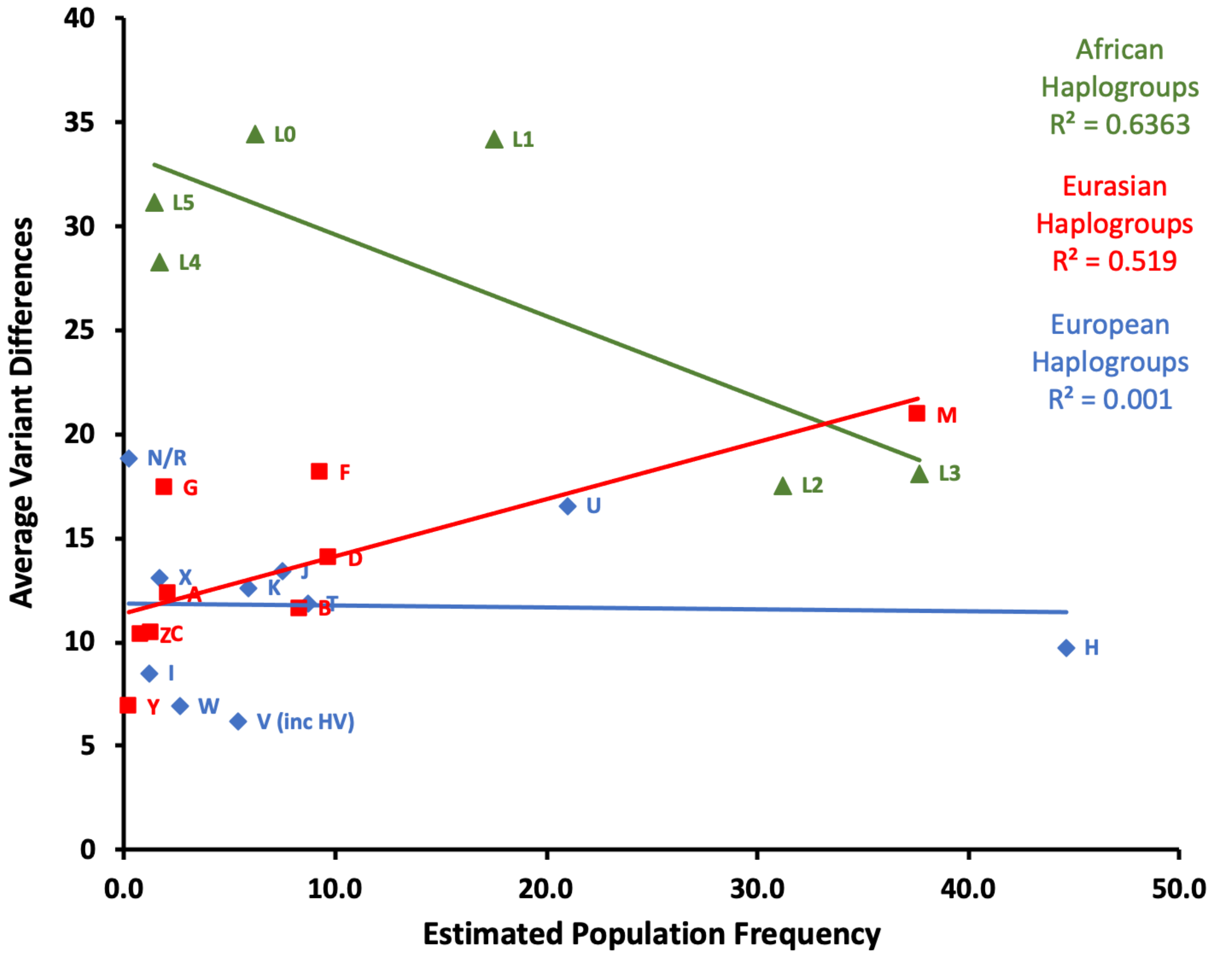

Appendix figure S4 | mtDNA haplogroup diversity versus estimated population frequency. Plot of the average variant differences (y) of the major haplogroups in each population group (African, European and Eurasian, **Datasets EV3a-c**) versus estimated population frequency (y). Shown is the correlation coefficient (R^2).

Appendix figure S5

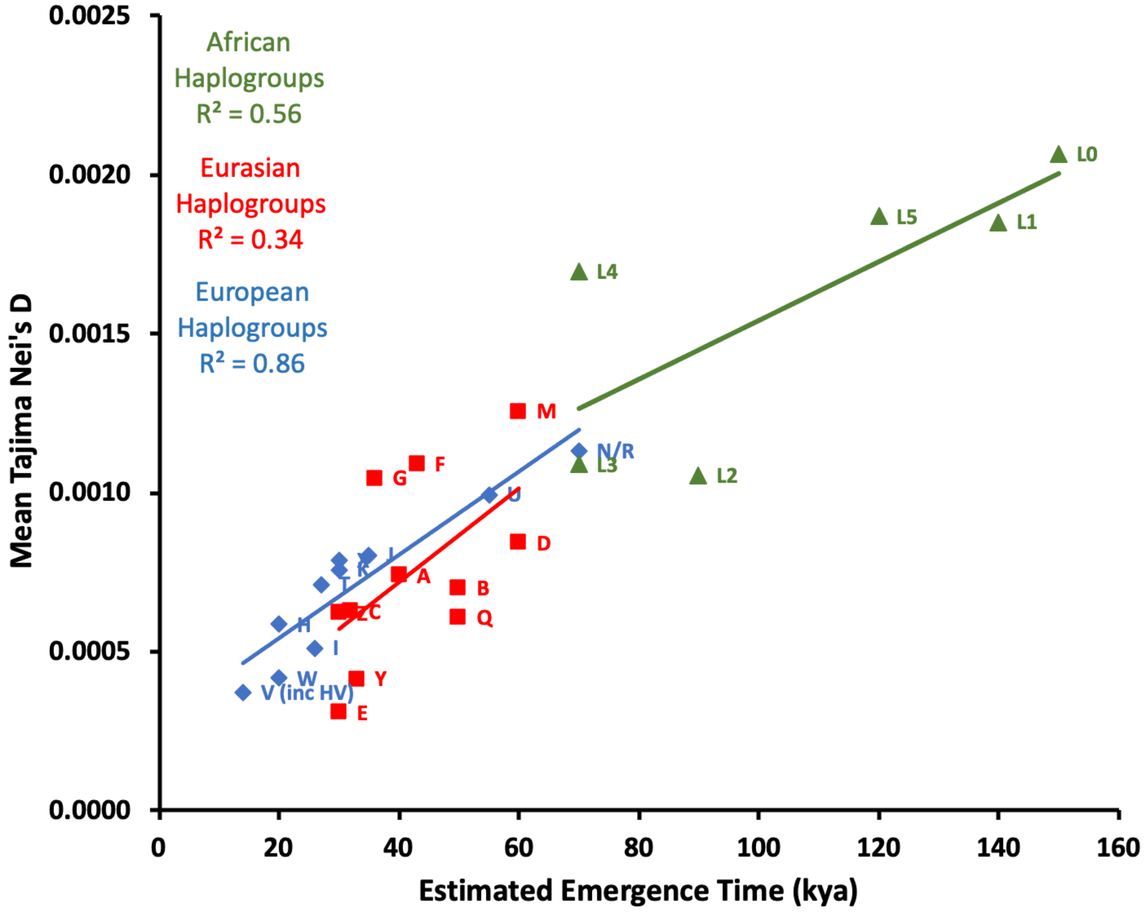

Appendix figure S5 | mtDNA haplogroup diversity versus estimated evolutionary age. Plot of the estimated evolutionary age (x) of the major haplogroups in each population group (African, European and Eurasian, **Datasets EV3a-c**) versus mean Tajima Nei's distance estimates for randomly selected pairs of sequences (y). Shown is the correlation coefficient (R^2).

Appendix figure S6

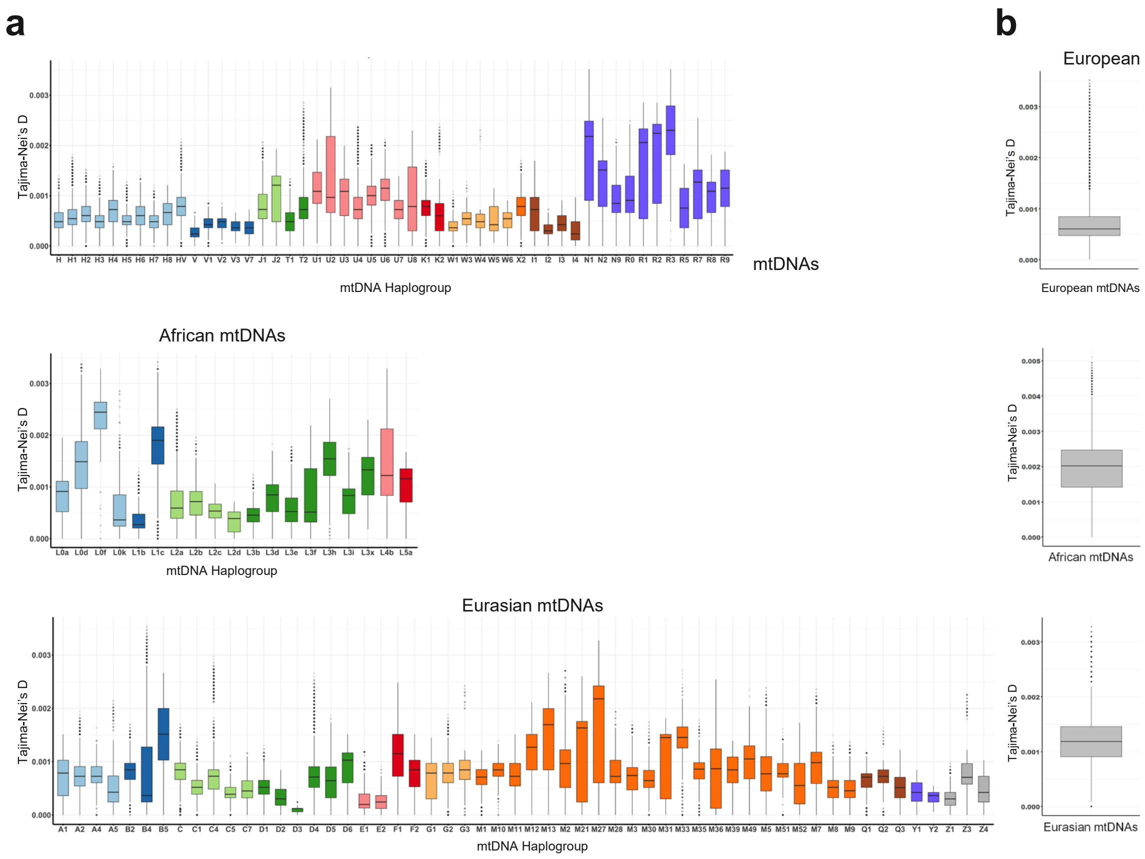

Appendix figure S6 | MtDNA sequence divergence within common European, African and Eurasian haplogroup sub-clades.

a, Boxplots showing estimated mtDNA pairwise sequence divergence within the commonest haplogroup sub-clades of each study population, where upper=European, middle=African and lower=Eurasian (**Datasets EV3a-c**). The intra-haplogroup mtDNA sequence divergence of each population differs significantly (one-way ANOVA in each population was p<2.2x10-16). **b,** Boxplots showing estimated mtDNA pairwise sequence divergence of any two random sequences from within each study population (upper=European, middle=African and lower=Eurasian, **Datasets EV3a-c**). All boxplots show median, 25th and 75th percentile, with whiskers indicating 1.5 upper and lower interquartile range. Dots indicate outliers. Data are based 7,655 European, 3,688 African and 6,857 Eurasian mtDNA sequences. Population groups were defined by mtDNA haplogroup (**Datasets EV3a-c**). Tajima-Nei's genetic distance model (Tajima-Nei's D) was used to investigate sequence divergence (SMaterials), where Tajima-Nei's D 0.00006=1 variant difference.

Appendix table S1| Mean mtDNA variant differences between unmatched and haplogroup matched sequence pairs.
a) Table showing the mean number of variant differences (with 95% CI) between randomly selected unmatched European, African and Eurasian mtDNA pairs (greyed) and the equivalent when sequence pairs are selected from within mtDNA haplogroup subclades in each population (**Datasets EV3a-c**). Arrows indicate either an intra-haplogroup subclade increase or decrease in variant differences relative to unmatched mtDNAs and P is the comparison of intra-haplogroup matched versus unmatched by Mann Whitney U, dashes indicate no significant change. Also shown is the number of sequences (and percentage frequency) used to make the divergence estimates and the maximum number variant differences observed between randomly paired sequences in each haplogroup sub-clade. b) Table showing the mean number of non-synonymous variant differences (with 95% CI) between randomly selected unmatched mtDNA from all datasets (African, European and Eurasian combined) and paired permutations of population group (i.e. African and European mtDNAs, **Datasets EV3d**). Nei-Gojobori model was used to investigate non-synonymous variant differences.

a)

			intra-group diversity					
		**% Frequency within dataset**	**No. of sequences for divergence estimate**	**% of dataset**	**Mean Variant Difference (95% CI)**	**Increased (▲) decreased (▼) or not significantly changed (-) compared to total dataset**	**Matched versus Total Dataset P**	**Maximum Variant Differences**
	Total European Dataset	-	7655	-	11.28 (11.16-11.40)	-	-	59
**Major European Haplogroup Subclades**	*H*	*1.4%*	*110*	1.4%	8.92 (8.24-9.60)	▼	*3.3E-08*	*23.20*
	H1	*18.0%*	*1378*	18.0%	9.8 (9.60-9.99)	▼	*4.1E-34*	*30.30*
	H2	*4.4%*	*339*	4.4%	17.24 (11.87-22.60)	-	*1.0E+00*	*39.00*
	H3	*5.5%*	*420*	5.5%	8.15 (7.87-8.44)	▲	*9.7E-67*	*23.25*
	H4	*2.2%*	*166*	2.2%	11.85 (11.14-12.57)	-	*1.0E+00*	*27.23*
	H5	*4.8%*	*366*	4.8%	8.6 (8.29-8.90)	▲	*2.3E-45*	*21.18*
	H6	*2.3%*	*179*	2.3%	10.12 (9.51-10.72)	▼	*1.4E-02*	*23.20*
	H7	*1.6%*	*125*	1.6%	7.86 (7.37-8.35)	▲	*8.8E-25*	*20.17*
	H8	*0.4%*	*30*	0.4%	10.65 (9.03-12.27)	-	*1.0E+00*	*20.18*
	HV	*3.0%*	*228*	3.0%	13.5 (12.86-14.15)	▲	*8.9E-09*	*35.33*
	V	*0.8%*	*61*	0.8%	4.27 (3.72-4.81)	▼	*3.4E-33*	*11.08*

	V1	*1.0%*	*75*	1.0%	7.36 (6.72-7.99)	▼	*1.7E-17*	*14.12*
	V2	*0.3%*	*21*	0.3%	7.56 (6.35-8.78)	▼	*3.5E-04*	*14.12*
	V3	*0.2%*	*17*	0.2%	6.1 (5.06-7.15)	▼	*1.8E-06*	*11.08*
	V7	*0.2%*	*18*	0.2%	6.16 (4.84-7.47)	▼	*3.3E-05*	*12.10*
	J1	*5.9%*	*453*	5.9%	13.26 (12.74-13.77)	▲	*4.3E-11*	*34.38*
	J2	*1.4%*	*109*	1.4%	15.93 (14.41-17.46)	▲	*1.5E-06*	*32.28*
	T1	*2.6%*	*197*	2.6%	8.32 (7.78-8.85)	▼	*1.6E-19*	*22.18*
	T2	*6.2%*	*478*	6.2%	12.45 (12.00-12.91)	▲	*6.7E-05*	*47.50*
	U1	*0.4%*	*32*	0.4%	19.18 (16.86-21.49)	▲	*8.5E-06*	*35.32*
	U2	*1.6%*	*120*	1.6%	22.15 (19.78-24.52)	▲	*2.5E-13*	*52.53*
	U3	*0.6%*	*48*	0.6%	16.6 (14.51-18.69)	▲	*4.4E-04*	*33.28*
	U4	*2.6%*	*198*	2.6%	13.74 (12.69-14.80)	▲	*4.7E-04*	*39.38*
	U5	*7.9%*	*608*	7.9%	16.44 (16.03-16.86)	▲	*8.3E-90*	*33.45*
	U6	*2.7%*	*209*	2.7%	18.32 (17.54-19.09)	▲	*8.6E-42*	*35.32*
	U7	*0.4%*	*31*	0.4%	11.61 (9.87-13.34)	-	*1.0E+00*	*23.18*
	U8	*0.5%*	*39*	0.5%	15.53 (12.05-19.01)	-	*1.0E+00*	*38.33*
	K1	*7.0%*	*539*	7.0%	12.74 (12.40-13.08)	▲	*7.0E-13*	*28.27*
	K2	*1.5%*	*114*	1.5%	10.02 (8.93-11.10)	-	*1.0E+00*	*40.38*
	W1	*0.9%*	*71*	0.9%	6.38 (5.64-7.12)	▼	*1.2E-18*	*18.15*
	W3	*0.3%*	*24*	0.3%	9.52 (8.01-11.02)	-	*1.0E+00*	*21.17*
	W4	*0.1%*	*11*	0.1%	12.53 (5.75-19.30)	-	*1.0E+00*	*38.37*
	W5	*0.2%*	*16*	0.2%	8.41 (6.05-10.76)	-	*1.0E+00*	*19.15*
	W6	*0.2%*	*13*	0.2%	8.76 (6.98-10.54)	-	*8.4E-01*	*15.12*
	X2	*1.9%*	*142*	1.9%	13.11 (12.30-13.93)	▲	*1.2E-03*	*28.25*
	I1	*0.6%*	*45*	0.6%	11.01 (9.23-12.79)	-	*3.8E+01*	*28.25*

	I2	*0.5%*	*36*	0.5%	5.57 (4.84-6.29)	▼	*8.8E-16*	*12.10*
	I3	*0.2%*	*18*	0.2%	7.26 (5.76-8.75)	▼	*3.1E-03*	*15.12*
	I4	*0.2%*	*16*	0.2%	5.12 (3.37-6.87)	▼	*2.5E-04*	*16.13*
	N1	*1.1%*	*82*	1.1%	30.48 (27.37-33.59)	▲	*4.1E-18*	*58.65*
	N2	*0.3%*	*22*	0.3%	21.44 (17.12-25.76)	▲	*7.6E-03*	*42.43*
	N9	*1.7%*	*129*	1.7%	15.1 (13.86-16.34)	▲	*9.2E-07*	*35.33*
	R0	*1.5%*	*116*	1.5%	17.26 (15.76-18.75)	▲	*1.4E-10*	*41.42*
	R1	*0.4%*	*30*	0.4%	26.06 (20.70-31.43)	▲	*4.1E-04*	*47.47*
	R2	*0.3%*	*23*	0.3%	29.08 (22.88-35.29)	▲	*5.9E-04*	*47.48*
	R3	*0.3%*	*20*	0.3%	36.98 (31.74-42.23)	▲	*5.0E-07*	*58.65*
	R5	*0.3%*	*25*	0.3%	12.86 (10.11-15.60)	-	*1.0E+00*	*27.23*
	R7	*0.2%*	*18*	0.2%	18.38 (13.28-23.48)	-	*7.2E-01*	*42.42*
	R8	*0.8%*	*65*	0.8%	16.67 (15.17-18.17)	▲	*7.7E-08*	*30.27*
	R9	*0.3%*	*25*	0.3%	18.6 (15.65-21.55)	▲	*3.0E-03*	*31.28*

**Major African Haplogroup Subclades**	Total African Dataset	-	3688	-	32.80 (32.33-33.27)	-	-	85
	L0a	8.6%	318	8.6%	13.29 (12.61-13.97)	▼	1.2E-197	32.63
	L0d	14.7%	543	14.7%	23.68 (22.74-24.61)	▼	1.9E-51	56.17
	L0f	0.4%	16	0.4%	37.78 (31.86-43.70)	-	1.0E+00	54.75
	L0k	2.9%	106	2.9%	9.23 (7.73-10.73)	▼	6.8E-55	47.53
	L1b	6.9%	255	6.9%	5.77 (5.34-6.20)	▼	4.5E-16	22.65
	L1c	12.2%	449	12.2%	28.82 (27.84-29.81)	▼	7.8E-09	56.87
	L2a	17.2%	636	17.2%	11.71 (11.20-12.23)	▼	3.8E-16	41.75

	L2b	2.1%	79	2.1%	11.06 (9.78-12.33)	▼	4.7E-50	32.63
	L2c	2.8%	104	2.8%	8.77 (8.17-9.36)	▼	3.1E-141	17.85
	L2d	0.4%	16	0.4%	5.84 (4.31-7.37)	▼	1.3E-15	11.88
	L3b	3.9%	142	3.9%	7.41 (6.91-7.92)	▼	4.3E-223	20.60
	L3d	5.7%	212	5.7%	12.66 (11.75-13.56)	▼	3.1E-118	30.48
	L3e	13.5%	497	13.5%	9.34 (8.87-9.81)	▼	6.6E-15	29.53
	L3f	4.3%	160	4.3%	13.11 (11.63-14.58)	▼	1.3E-58	36.53
	L3h	1.2%	44	1.2%	25.06 (22.41-27.72)	▼	7.2E-05	45.07
	L3i	0.4%	14	0.38%	13.11 (9.35-16.87)	▼	3.1E-06	28.93
	L3x	0.6%	21	0.57%	20.52 (17.30-23.74)	▼	1.1E-05	38.32
	*L4b**	0.9%	33	0.9%	22.77 (18.23-27.31)	▼	5.1E-03	54.77
	*L5a***	0.7%	27	0.7%	16.79 (14.02-19.55)	▼	4.3E-10	27.87

**Major Eurasian Haplogroup Subclades**	Total Eurasian Dataset	-	6857	-	20.12 (19.95-20.28)	-	-	59
	A1	0.2%	14	0.2%	12.02 (8.83-15.22)	▼	1.4E-02	25.20
	A2	4.1%	281	4.1%	12.51 (11.96-13.06)	▼	2.5E-80	32.32
	A4	0.8%	56	0.8%	12.31 (11.31-13.30)	▼	4.1E-20	27.25
	A5	1.0%	66	1.0%	8.59 (7.09-10.08)	▼	2.6E-21	35.67
	B2	2.0%	136	2.0%	13.81 (13.18-14.44)	▼	8.7E-40	28.27
	B4	11.6%	792	11.6%	11.49 (10.78-12.19)	▼	6.6E-93	58.87
	B5	1.0%	66	1.0%	24.72 (22.29-27.16)	▲	2.5E-02	44.45
	C	2.2%	153	2.2%	13.32 (12.48-14.16)	▼	2.1E-32	28.30
	C1	3.2%	220	3.2%	8.68 (8.26-9.09)	▼	2.7E-141	23.83

	C4	5.6%	382	5.6%	11.34 (10.83-11.85)	▼	8.5E-118	49.62
	C5	1.5%	105	1.5%	6.65 (6.07-7.24)	▼	4.7E-73	18.40
	C7	0.8%	57	0.8%	8.03 (7.08-8.98)	▼	8.7E-31	19.30
	D1	3.6%	250	3.6%	8.61 (8.21-9.00)	▼	1.2E-163	21.63
	D2	0.9%	62	0.9%	5.42 (4.58-6.27)	▼	3.5E-41	14.10
	D3	0.4%	26	0.4%	1.66 (1.26-2.06)	▼	1.7E-40	4.03
	D4	14.0%	963	14.0%	12.26 (11.95-12.57)	▼	7.3E-274	44.30
	D5	3.2%	216	3.2%	10.48 (9.69-11.27)	▼	1.4E-60	31.12
	D6	0.4%	24	0.4%	14.98 (12.52-17.43)	▼	2.3E-02	25.22
	E1	2.5%	169	2.5%	4.75 (4.22-5.28)	▼	6.5E-121	19.70
	E2	0.9%	65	0.9%	4.31 (3.68-4.94)	▼	9.1E-55	15.17
	F1	1.9%	131	1.9%	18.26 (16.93-19.60)	-	4.3E-01	41.40
	F2	0.2%	17	0.2%	12.9 (10.23-15.58)	▼	3.9E-03	25.22
	G1	1.2%	82	1.2%	11.17 (9.87-12.47)	▼	8.9E-21	24.20
	G2	1.9%	132	1.9%	13.35 (12.49-14.21)	▼	6.2E-30	31.30
	G3	0.4%	25	0.4%	14.98 (11.88-18.09)	-	1.9E-01	40.35
	M1	1.8%	125	1.8%	11.56 (10.90-12.21)	▼	6.2E-52	20.15
	M10	0.5%	32	0.5%	13.83 (12.43-15.24)	▼	4.4E-08	23.18
	M11	0.4%	28	0.4%	12.84 (11.02-14.66)	▼	1.0E-06	25.22
	M12	0.6%	42	0.6%	19.25 (16.64-21.87)	-	1.0E+00	35.30
	M13	0.6%	40	0.6%	23.4 (19.73-27.07)	-	1.0E+00	44.40
	M2	2.3%	160	2.3%	15.8 (14.48-17.12)	▼	9.7E-08	45.08
	M21	0.6%	38	0.6%	20.21 (16.16-24.26)	-	1.0E+00	43.40
	M27	2.1%	142	2.1%	27.85 (25.15-30.54)	▲	5.8E-06	54.53
	M28	0.7%	51	0.7%	12.96 (11.26-14.66)	▼	3.9E-09	32.27

	M3	1.9%	129	1.9%	11.52 (10.58-12.47)	▼	7.3E-35	29.18
	M30	1.1%	73	1.1%	11.06 (10.07-12.05)	▼	5.1E-27	29.02
	M31	0.4%	27	0.4%	17.51 (13.54-21.48)	-	1.0E+00	30.27
	M33	0.7%	50	0.7%	23.11 (20.97-25.24)	-	4.8E-01	45.23
	M35	0.8%	56	0.8%	13.48 (12.18-14.77)	▼	1.8E-12	29.68
	M36	0.6%	38	0.6%	12.06 (8.64-15.48)	▼	2.7E-03	42.40
	M39	0.4%	27	0.4%	13.91 (11.81-16.00)	▼	2.6E-04	23.17
	M49	0.4%	30	0.4%	16.18 (12.85-19.50)	-	1.0E+00	39.15
	M5	2.1%	144	2.1%	13.59 (12.39-14.79)	▼	3.1E-18	39.73
	M51	0.4%	26	0.4%	12.73 (9.99-15.47)	▼	9.5E-04	26.85
	M52	0.5%	37	0.5%	10.14 (7.74-12.53)	▼	5.1E-08	28.83
	M7	7.9%	542	7.9%	14.48 (13.91-15.04)	▼	2.1E-61	39.30
	M8	0.6%	44	0.6%	8.49 (7.24-9.74)	▼	1.7E-20	24.87
	M9	2.6%	180	2.6%	8.13 (7.60-8.67)	▼	3.4E-105	22.70
	Q1	1.6%	113	1.6%	10.33 (9.50-11.17)	▼	1.2E-43	19.38
	Q2	0.4%	26	0.4%	11.75 (9.53-13.98)	▼	5.7E-06	27.22
	Q3	0.2%	17	0.2%	8.56 (6.42-10.70)	▼	6.4E-07	20.35
	Y1	0.3%	20	0.3%	7.37 (5.92-8.82)	▼	1.8E-11	14.12
	Y2	0.2%	13	0.2%	5.74 (4.57-6.90)	▼	3.5E-10	9.07
	Z1	1.0%	66	1.0%	4.93 (4.25-5.61)	▼	1.2E-51	16.12
	Z3	0.5%	36	0.5%	13.41 (10.71-16.11)	▼	1.4E-03	37.55
	Z4	0.2%	15	0.2%	7.79 (5.52-10.07)	▼	2.3E-06	17.13

b)

African, European and Eurasian mtDNAs	-	18,200	-	19.64 (19.5-19.9)	-	-	85
European and African mtDNAs	-	11,343	63.3%	24.92 (24.7-25.2)	▲	3.8E-302	85
European and Eurasian mtDNAs	-	14,512	79.7%	18.22 (18.1-18.3)	▼	4.9E-46	55
African and Eurasian mtDNAs	-	10,454	57.4%	23.76 (23.6-24.0)	▲	5.6E-298	80
