# Supplementary material for: Feasibility and impact of haplogroup matching for mitochondrial replacement treatment
Source: EMBO Rep. 2023 Aug 17;24(10):e54540. doi: 10.15252/embr.202154540 (PMC10561356; doi:10.15252/embr.202154540)
Supplement: Supplementary file 8 [file EMBR-24-e54540-s001.pdf]

EMBO reports

Feasibility and impact of haplogroup matching for mitochondrial replacement treatment

Yuko Takeda, Louise Hyslop, Meenakshi Choudhary, Fiona Robertson, Angela Pyle, Ian Wilson, Mauro Santibanez-Koref, Douglass Turnbull, Mary Herbert, and Gavin Hudson
DOI: 10.15252/embr.202154540

Corresponding author(s): Gavin Hudson (Gavin.Hudson@ncl.ac.uk) , Mary Herbert (mary.herbert@newcastle.ac.uk)

Review Timeline:

Submission Date:	17th Dec 21
Editorial Decision:	7th Feb 22
Revision Received:	19th Jun 23
Editorial Decision:	29th Jun 23
Revision Received:	3rd Jul 23
Accepted:	26th Jul 23

Editor: Deniz Senyilmaz Tiebe

Transaction Report:

(Note: With the exception of the correction of typographical or spelling errors that could be a source of ambiguity, letters and reports are not edited. Depending on transfer agreements, referee reports obtained elsewhere may or may not be included in this compilation. Referee reports are anonymous unless the Referee chooses to sign their reports.)

1st Editorial Decision **7th Feb 2022**

Dear Dr. Hudson,

Thank you for the submitting your manuscript to EMBO Reports. We have now received three referee reports, whose reports are copied below. Additionally, we sought advice from an external expert in the field whose opinion we trust.

I apologize for this unusual delay in getting back to you, which was due to the delay in to receiving the referee reports and the protracted internal discussions.

We concur with the referees that the manuscript poses a very interesting view on the necessity of intra-haplogroup matching between the egg donor and the patient undergoing mitochondrial replacement therapy (MRT). However, referees and also the advisor raise significant concerns. In particular, referee #1 and the advisor find that the study currently does not provide sufficient experimental evidence supporting that mixing mtDNA haplotypes does not elicit any long-term complications. The additional experimentation to show this would clearly be outside the scope of a realistic revision. In light of this, we concluded that, the manuscript is not suitable for publication in the Article section of EMBO Reports. Rather, we consider it better-suited for our Commentary section, where opinion pieces supported by preliminary analysis are published. Should that be of interest for you, we would like to invite you to submit a revised manuscript. Please address all referee concerns in a complete point-by-point response. Regarding point 1 of referee #2, we recommend refraining from a definitive conclusion given the nature of the supporting evidence.

Moreover, the manuscript needs to be significantly re-formatted given the strict format requirements of the Commentary section and some of the figures need to be removed as the referees considered them not fit for publication. In particular,

- Please reduce the number of main figures to around 2. As per the comments of referee #1 (1st paragraph), we propose removing the figures demonstrating the impact of haplogroup matching on the availability of egg donors (Figure 1) and geographical, ethnical and populational distribution of haplogroups (S Figures 1 to 5). These analyses can be mentioned in the text. We also propose moving S Figure 6 to the main figures as a panel for one of the main figures. Should you wish the removed figures/analyses to be publicly available, we recommend posting the manuscript to a recognized preprint server (e.g. bioRxiv) and citing the post in the manuscript.
- Please reduce the word count to 5000 by reframing conclusions. We trust this can be achieved without losing key substance.
- Please reduce the number of references to around 30.

I look forward to seeing a revised version of your manuscript when it is ready. Please let me know if you would like to discuss anything further.

Kind regards,

Deniz Senyilmaz Tiebe

Deniz Senyilmaz Tiebe, PhD
Scientific Editor
EMBO Reports

Referee #1:

Title: Feasibility and impact of haplogroup matching for mitochondrial replacement therapy.

In this manuscript, Takeda et al., present their data on estimated mtDNA haplogroup frequencies in the UK population and conclude that matching donor mtDNA haplogroup to patients with mtDNA disease will limit the availability of egg donors for IVF+MRT. This conclusion is so obvious and anticipated by just common sense and I don't think it required any special investigation. So, I am not sure if this manuscript brings any novelty or advances our knowledge.
Authors do not provide any solution to this bottleneck in MRT except arguing that mismatching of mtDNA haplotypes may not be detrimental for children born following MRT. These speculations were based on arguments that mixing of mtDNA and nuclear DNA from divergent populations during natural human reproduction does not cause any known health issues in children. However, mixing of mtDNA and nuclear DNA during natural fertilization is quite different than after MRT. Natural reproduction combines maternal mtDNA with maternal and paternal nuclear genomes. So, there is a match between maternal nuclear and mitochondrial genomes. In contrast, MRT combines unrelated donor mtDNA with maternal and paternal nuclear DNA.
If mtDNA haplotype matching is not necessary, authors should provide experimental evidence demonstrating that MRT offspring generated from unmatched (distant) mtDNA haplotypes do not display any long-term health complications.
There is no sufficient data suggesting that donor oocyte freezing is detrimental for MRT. I agree that suboptimal egg freezing procedures were problematic for IVF in the past. But recent developments in oocyte vitrification protocols suggest that frozen/thawed oocytes are comparable to fresh oocytes in clinical IVF pregnancy outcomes. There are large banks of vitrified

oocytes available worldwide that should make mtDNA haplotype matching feasible.

Referee #2:

This work addresses a very important issue, for all women carrying a pathogenic mutation in their mitochondrial DNA (mtDNA) and would like to conceive a healthy child by means of assisted reproduction with mitochondrial replacement. While technically possible, the question remains open whether there is a need to match donor mtDNA with the recipient mtDNA to maintain the "mitochondrial-nuclear genome crosstalk". The need for matching would markedly limit the availability of donor eggs. The authors concluded that intra-haplogroup variability is relatively large and questions the benefit of matching especially for rare haplogroups.
The manuscript is an important contribution to the field and contains a large amount of genetic information gathered from various databases clearly summarized in figures. It is well written and I have only a few minor comments/questions.
and I have only a few minor comments.
1)This might be an oversimplification, but since this article is expected to a have practical impact. What is the bottom line in plain text? Generally, do you recommend or not haplotype matching? This should also appear in the abstract

2)Do the haplogroups always match the same ethnicity? i.e. are there "whites" with African haplogroup and vice versa.

3)Do you propose that European donors are more "universal"? (like blood group 0). While African- or Eurasian donors would be more suitable for African- or Eurasian recipients?

4)Do you think haplogroups should be redefined in some way according to your data?

5)There are various reports about prevalence of certain conditions "autism etc etc " in certain haplogroups. Should these be excluded form donorship?

Referee #3:

the authors address an important issue, haplogroup matching for MRT. The authors point out that haplogroup matching is somewhat arbitrary, and does not take into account all sequence variation in the genome, which would be a more accurate measure of genetic differences. Genotyping of haplogroups is somewhat archaic, as it emerged in a time when full genome sequencing was expensive and impractical. Nowadays, the sequencing of an entire mitochondrial genome is no longer prohibitive.
Using sequencing data from entire mtDNA, and thereby taking all sequence variation into account, the authors find that haplogroup matching in MRT reduces genetic differences, but not in all cases. They conclude that the universal application of haplogroup matching is not meaningful, and could in fact have the opposite effect of the intended reduction of genetic differences. The authors also look specifically at nonsynonymous variants, and find no benefit of haplogroup matching. This is perhaps the strongest argument of the article. The authors quantify the estimated delay caused by matching. The authors base their calculations not only on the % haplogroup in a population, but also consider the % women who are interested and complete a donation cycle.
The authors also question the need for minimizing sequence divergence given the functional importance of genetic recombination in reproduction.
The reviewer is clearly supportive of publication, with some minor modifications.

Page 4: Regarding the observation that vitrified donor eggs may be more difficult to use, this could a technical issue that will go away, and can not be a primary reason for not doing haplogroup matching.
While an obvious solution to the problem are growing donor egg banks, that in principle would allow purchase and shipment of oocytes from any place in the world, and from any country and ethnicity, procuring them may be not simple, and local egg donors will remain more likely. The authors could discuss this some more. There is no discussion of egg banks. The article appears to be largely tailored to the situation/legal framework? of the center of the authors.

The authors quantify the estimated delay caused by matching. The authors base their calculations not only on the % haplogroup in a population, but also consider the % women who are interested and complete a donation cycle.

The section on page 8 about MRT across ancestries is counterintuitive and requires some more explanation why European donation to an African recipient reduces, rather than increases genetic differences as opposed to random donation within the group (clarify without matching versus with haplogroup matching).

The authors do not discuss mitochondrial sequence divergence in the context of heteroplasty between normal mtDNA species

after MRT. Authors should clarify if they rely on that this technical problem is going to be solved (and perhaps state how), or comment on the consequences of such mixed mtDNA populations.

One issue that the comparison of total sequence differences has, and even of the nonsynonymous variants, is that we do not know how to weigh these differences. The authors should comment on the knowledge we have about the functional relevance of mtDNA variants (or lack thereof). The authors could also discuss the need for functional interrogation of the mtDNA variants, e.g. through the ability to modify mtDNA in cell lines. The absence of such knowledge does make any meaningful matching a well-meaning intent, but one that could be misinformed.

Typo page 3: the mitochondrial genome exists in... (not exits)

Response to Reviewers Comments.

Referee 1.
We thank the reviewer for taking the time to review our manuscript.

Response to comments:
Q1) In this manuscript, Takeda et al., present their data on estimated mtDNA haplogroup frequencies in the UK population and conclude that matching donor mtDNA haplogroup to patients with mtDNA disease will limit the availability of egg donors for IVF+MRT. This conclusion is so obvious and anticipated by just common sense and I don't think it required any special investigation. So, I am not sure if this manuscript brings any novelty or advances our knowledge.

We agree with the Referee, that the negative impact of haplogroup matching on donor availability may be intuitive to some. However, based on the published literature, the shortage of egg donors is not fully appreciated by advocates of haplogroup matching. Moreover, the potential impact of haplogroup matching on MRT waiting times has not previously been quantified. Up until to now, we could only say that there would be a delay in treatment. The finding that women belonging to rare haplogroups may have to wait for a substantial fraction of their reproductive lives before finding a haplogroup matched donor will help to inform policy makers and regulators. To emphasise this point, we have amended the text introduction (**lines 123- 130** and **134-136**) and the discussion (**lines 317-327** and **lines 370-373**).

Q2) The authors do not provide any solution to this bottleneck in MRT except arguing that mismatching of mtDNA haplotypes may not be detrimental for children born following MRT. These speculations were based on arguments that mixing of mtDNA and nuclear DNA from divergent populations during natural human reproduction does not cause any known health issues in children. However, mixing of mtDNA and nuclear DNA during natural fertilization is quite different than after MRT. Natural reproduction combines maternal mtDNA with maternal and paternal nuclear genomes. So, there is a match between maternal nuclear and mitochondrial genomes. In contrast, MRT combines unrelated donor mtDNA with maternal and paternal nuclear DNA.

We have now included elaborated on the potential use of egg banks to increase availability of mtDNA diversity of donated eggs (**lines 317-327**). However, until current limitations associated with the use of vitrified donor eggs can be overcome, clinical MRT relies largely on donated eggs from the local population. (Please see also response to Q4).
We agree with the reviewer that MRT differs from natural reproduction, and we have elaborated on the relevance of this in **lines 346-359**. We have also highlighted the potential for mitochondrial-independent effects of MRT in the concluding paragraph (**lines 378-381**).

Q3) If mtDNA haplotype matching is not necessary, authors should provide experimental evidence demonstrating that MRT offspring generated from unmatched (distant) mtDNA haplotypes do not display any long-term health complications.

Our investigation aimed to determine whether mtDNA sequence divergence would be reduced by haplogroup matching. We did not set out to investigate the long-term health implications and would like to point out that the experimental evidence requested by the referee may take some decades.

Q4) There is no sufficient data suggesting that donor oocyte freezing is detrimental for MRT. I agree that suboptimal egg freezing procedures were problematic for IVF in the past. But recent developments in oocyte vitrification protocols suggest that frozen/thawed oocytes are comparable to fresh oocytes in clinical IVF pregnancy outcomes. There are large banks of vitrified oocytes available worldwide that should make mtDNA haplotype matching feasible.

We suspect that the referee is referring to an effect of vitrification on embryo survival and development. We apologise for not making it clear that the detrimental effect to we refer to pertains specifically to MRT the efficacy of MRT. While we agree that the possibility to partner with an established egg bank may help to alleviate the scarcity of donor eggs, our preclinical research indicated that the fraction of mtDNA carried over was generally higher when mitochondrial donor eggs were vitrified (Hyslop *et al*, Nature, 2016, see Figure 4c). For this reason, the current clinical pathway involves the use of freshly harvested donor eggs and vitrified patient eggs. We have elaborated on this issue and the potential use of egg banks in **lines 317-327**.

<u>Referee 2.</u>

We are encouraged by the positive and supportive comments of Referee 2, and we are pleased that the referee recognises that the effect of haplogroup matching is an important issue that warrants publication.

Response to comments:

Q1) This might be an oversimplification, but since this article is expected to a have practical impact. What is the bottom line in plain text? Generally, do you recommend or not haplotype matching? This should also appear in the abstract.

We agree with the reviewer and have edited the concluding remarks to emphasise our position, **lines 373-376** “Given the likely impact on the availability of donor eggs, we propose that haplogroup matching is not warranted solely on the basis of reducing sequence divergence between donor/recipient pairs.” We have emphasised our conclusions in the abstract, **lines 64-65**) “These findings indicate that haplogroup matching would restrict the availability of MRT, without necessarily reducing mtDNA sequence divergence between donor/recipient pairs.”

Q2) Do the haplogroups always match the same ethnicity? i.e. are there "whites" with African haplogroup and vice versa.

Although haplogroup designation is intrinsically linked to ethnic ancestry (**lines 109-112**), increased mobility, migration and admixture, and the nature by which ethnicity (which can

be distinct from ancestry) is reported has blurred these boundaries somewhat. Indeed, our data and that of others (e.g. https://www.eupedia.com/genetics/, Cardena *et al*., 2013 PLoS One, Lee *et al*., 2011 BMC Proc and Ely *et al*.,, 2006 BMC Biology) suggest that mtDNA haplogroup is around 90-95% accurate in predicting ethnicity.

Q3) Do you propose that European donors are more "universal"? (like blood group 0). While African- or Eurasian donors would be more suitable for African- or Eurasian recipients?

Based on our data, the idea of a 'universal mtDNA donor' seems unlikely. Our data shows that there is significant diversity between mtDNAs from the same population (as expected) but also within haplogroups and haplogroup sub-clades.

Q4) Do you think haplogroups should be redefined in some way according to your data?

mtDNA haplogroup structure is a construct defined by currently available mtDNA sequence data. As more individuals are sequenced, their data can be used to refine our definition of haplogroups.

Q5) There are various reports about prevalence of certain conditions "autism etc " in certain haplogroups. Should these be excluded form donorship?

Although it is true that common mtDNA variation is associated with common disease risk, very few associations (with the exception of say Parkinsons and Alzheimers disease, multiple sclerosis, reviewed in Chinnery *et al*., 2018 Front Neurosci) have ever been replicated or validated in multiple cohorts. We have included as sentence in the discussion to highlight this, **lines 373-376.** Our current clinical practice (approved by the HFEA) is to test donor mtDNA for known pathogenic variants.

Referee 3.

We are encouraged by the positive and supportive comments of Referee 3 and we are pleased that the referee is supportive of publication.

Response to comments:

Q1) Page 4: Regarding the observation that vitrified donor eggs may be more difficult to use, this could a technical issue that will go away, and cannot be a primary reason for not doing haplogroup matching.

Our preclinical research indicated that the fraction of mtDNA carried over was increased when mitochondrial donor eggs were vitrified (Hyslop *et al*., Nature, 2016, see Figure 4c). Thus, the current clinical pathway involves the use of freshly harvested donor eggs and vitrified patient eggs. We would like to share the reviewer's optimism that it will become possible to use vitrified donor eggs in the future and we continue to investigate this. The ability to use vitrified donor eggs would potentially reduce the waiting time for under-

represented haplogroups but would be unlikely to alleviate the problem for intrinsically rare haplogroups. These points are now clarified in **lines 123-130** and **317-327**.

Q2) While an obvious solution to the problem are growing donor egg banks, that in principle would allow purchase and shipment of oocytes from any place in the world, and from any country and ethnicity, procuring them may be not simple, and local egg donors will remain more likely. The authors could discuss this some more. There is no discussion of egg banks. The article appears to be largely tailored to the situation/legal framework? of the center of the authors.

As above, the potential for using egg banks in the future is now discussed in **lines 317-327**.

Q3) The authors quantify the estimated delay caused by matching. The authors base their calculations not only on the % haplogroup in a population, but also consider the % women who are interested and complete a donation cycle.

The referee is correct, these calculations are based on our in-house egg donor programme and serve to underscore the considerable difficulty in obtaining donated eggs. The massive (94%) drop-off between expression of interest and completion of an egg donation cycle may not be obvious to those who not directly involved in the delivery of ART procedures.

Q4) The section on page 8 about MRT across ancestries is counterintuitive and requires some more explanation why European donation to an African recipient reduces, rather than increases genetic differences as opposed to random donation within the group (clarify without matching versus with haplogroup matching).

We have added a sentence and relevant references to help explain the reduced variant count when European haplogroup egg donors are used for the treatment of patients belonging to African haplogroups (**lines 274-276**).

Q5) The authors do not discuss mitochondrial sequence divergence in the context of heteroplasmy between normal mtDNA species after MRT. Authors should clarify if they rely on that this technical problem is going to be solved (and perhaps state how), or comment on the consequences of such mixed mtDNA populations.

We have included a sentence on potential effects of non-haplogroup defining variants on heteroplasmy for maternal mtDNA in the concluding paragraph (**lines 373-376**).

Q6) One issue that the comparison of total sequence differences has, and even of the nonsynonymous variants, is that we do not know how to weigh these differences. The authors should comment on the knowledge we have about the functional relevance of mtDNA variants (or lack thereof). The authors could also discuss the need for functional interrogation of the mtDNA variants, e.g. through the ability to modify mtDNA in cell lines. The absence of such knowledge does make any meaningful matching a well-meaning intent, but one that could be misinformed.

The reviewer makes a good point and we have now included a sentence on the gaps in our knowledge about the functional relevance of specific variants, or indeed combinations of variants in the concluding paragraph (**lines 373-381**)

Q7) Typo page 3: the mitochondrial genome exists in... (not exits)
Corrected.

1st Revision - Editorial Decision **29th Jun 2023**

Dear Gavin,
Dear Mary,

I just noticed that the decision letter I sent was missing a few items - my apologies for the oversight. Please below find the complete letter.

Kind regards,

Deniz
--

Dear Gavin,
Dear Mary,

Thank you for submitting your revised manuscript. I apologize for the delay in getting back. I have now read all the files, and my colleagues have performed the revision checks on the manuscript. Below are a few editorial points to address before I can accept the manuscript for publication in EMBO Reports.

Given the nature of the required revisions, and to ensure timely publication of this exciting manuscript, we believe that two weeks should be sufficient for resubmission (by 13.07). Please let me know if you are unable to meet this deadline.

- Having evaluated the revised manuscript and all figures in the editorial team, we decided that we would like to convert Figure 1 into EV Figure 1 (Expanded View Figure 1). Please update in-text callouts accordingly.
- Please rename the PDF file called 'Supplementary Figures' as Appendix. Please update the in-text callouts of the supplementary figures as Appendix Figure S1 etc. Of note, Appendix figures are not the same as EV Figures (for further information please see https://www.embopress.org/page/journal/14693178/authorguide#expandedview)
- We note that the author checklist was submitted, but it is incomplete - i.e. D73, D74 and D77 cells need to be filled in.
- Please remove 'Author Contributions' section from the manuscript.
- Please rename the Conflict of Interests section as 'Disclosure and competing interests statement'
- As per our format requirements, in the reference list, citations should be listed in alphabetical order and then chronologically, with the authors' surnames and initials inverted; where there are more than 10 authors on a paper, 10 will be listed, followed by 'et al.'. Please see https://www.embopress.org/page/journal/14693178/authorguide#referencesformat
- The list with supplementary materials on p. 13 should be deleted.
- The Paper Explained should be deleted.
- We note that Figure 3B is currently not called out in the text.
- We note that the author checklist was submitted, but it is incomplete - i.e. D73, D74 and D77 cells need to be filled in.
- Please resubmit the source data as one file per figure.
- Papers published in EMBO Reports include a 'synopsis' and 'bullet points' to further enhance discoverability. Both are displayed on the html version of the paper and are freely accessible to all readers. The synopsis includes a short standfirst summarizing the study in 1 or 2 sentences that summarize the paper (max 35 words) and are provided by the authors and streamlined by the handling editor. I would therefore ask you to include your synopsis blurb and 3-5 bullet points listing the key findings.
- In addition, please provide an image for the synopsis. This image should provide a rapid overview of the question addressed in the study but still needs to be kept fairly modest since the image size cannot exceed 550x400 pixels.
- Our production/data editors have asked you to clarify several points in the figure legends (see attached document). Please incorporate these changes in the attached word document and return it with track changes activated.

Thank you again for the opportunity to consider this manuscript. I am looking forward to receiving your minor revision!

Kind regards,

Deniz

Deniz Senyilmaz Tiebe, PhD
Editor
EMBO Reports

<u>*Editorial Notes/Changes*</u>

1) Having evaluated the revised manuscript and all figures in the editorial team, we decided that we would like to convert Figure 1 into EV Figure 1 (Expanded View Figure 1). Please update in-text callouts accordingly.
Changes:
Figure 1 renamed to Expanded View Figure 1
Figure 2 renamed to Figure 1
Figure 3 renamed to Figure 2
Figure 4 renamed to Figure 3

2) Please rename the PDF file called 'Supplementary Figures' as Appendix.
Please update the in-text callouts of the supplementary figures as Appendix Figure S1 etc.
Changes:
Supplementary Figures and Tables renamed to Appendix Figure S# and Appendix Table S#
Note we have moved Appendix Table S1 into the Appendix file.

3) We note that the author checklist was submitted, but it is incomplete - i.e. D73, D74 and D77 cells need to be filled in.
Changes:
Checklist completed.

4) Please remove 'Author Contributions' section from the manuscript.
Changes:
Removed Text

5) Please rename the Conflict of Interests section as 'Disclosure and competing interests statement'
Changes:
Renamed Text

6) As per our format requirements, in the reference list, citations should be listed in alphabetical order and then chronologically, with the authors' surnames and initials inverted; where there are more than 10 authors on a paper, 10 will be listed, followed by 'et al.'.
Changes:
Citations and reference list have been changed as per guidance (using EMBO Reports Harvard style).

7) The list with supplementary materials on p. 13 should be deleted.
Changes:
Text removed.

8) The Paper Explained should be deleted.
Changes:
Text removed.

9) We note that Figure 3B is currently not called out in the text.
Changes:
See lines 289 and 290

10) Please resubmit the source data as one file per figure.
Changes:
File separated and uploaded individually

11) Figure Legend Changes (note the new numbering used)

For Expanded View Figure 1, please define the number and the nature, i.e. biological or technical, of the replicates.

The graph shows estimated frequency (i.e. count) based on population data. The graph does not show biological or technical replicates. However, we have added the donor count used to make estimate frequencies. We are edited to the legend with the suggested changes:

c) Graph showing the estimated number of women per 100,000 that proceed to egg donation (red) in relation to those that are eligible to donate (blue) for each of the commonest mtDNA haplogroups identified across European populations (Based on 316 donors, SDatasets 1-2, and assuming the haplogroup of those who donate eggs is reflective of ancestry).

We have edited the legend to include the data description and suggested changes: *Data information: Bar graphs show mean and standard deviation.*

For Figure 1, Please define the error bars, e.g. SD and also clarify the number and the nature, i.e. biological or technical, of the replicates for panels A and C.

We have edited the legend to include the data description and suggested changes:

Data information: Boxplots show median, 25th and 75th percentile, with whiskers indicating 95th upper/lower interquartile range. Dots indicate outliers. Bar charts show mean, and standard deviation. Estimates (a-b) and counts (c) are based on 7,655 European, 3,688 African and 6,857 Eurasian mtDNA sequences. Population groups were defined by mtDNA haplogroup. Tajima-Nei's genetic distance model (Tajima-Nei's D) was used to derive sequence divergence, where Tajima-Nei's D 0.00006=1 variant difference.

For Figure 2, Please define the error bars, e.g. SD and also clarify the number and the nature, i.e. biological or technical, of the replicates for panels A and C.

We have edited the legend to include the data description and suggested changes:

Data information: Boxplots show median, 25th and 75th percentile, with whiskers indicating 95th upper/lower interquartile range. Dots indicate outliers. Bar charts show mean and standard deviation. Estimates (a-b) and counts (c) are based on 7,655 European, 3,688 African and 6,857 Eurasian mtDNA sequences. Population groups were defined by mtDNA haplogroup. Nei-Gojobori model was used to investigate non-synonymous variant differences.

2nd Revision - Editorial Decision 26th Jul 2023

Dear Gavin,
Dear Mary,

Thank you for submitting your revised manuscript. I have now looked at everything and all looks fine. Therefore, I am very pleased to accept your manuscript for publication in EMBO Reports!

Kind regards,

Deniz

--
Deniz Senyilmaz Tiebe, PhD
Scientific Editor
EMBO Reports
--

At the end of this email I include important information about how to proceed. Please ensure that you take the time to read the information and complete and return the necessary forms to allow us to publish your manuscript as quickly as possible.

As part of the EMBO publication's Transparent Editorial Process, EMBO reports publishes online a Review Process File to accompany accepted manuscripts. As you are aware, this File will be published in conjunction with your paper and will include the referee reports, your point-by-point response and all pertinent correspondence relating to the manuscript.

If you do NOT want this File to be published, please inform the editorial office within 2 days, if you have not done so already, otherwise the File will be published by default [contact: emboreports@embo.org]. If you do opt out, the Review Process File link will point to the following statement: "No Review Process File is available with this article, as the authors have chosen not to make the review process public in this case." Please note that the author checklist will still be published even if you opt out of the transparent process.

Thank you again for your contribution to EMBO Reports and congratulations on a successful publication. Please consider us again in the future for your most exciting work.

****

THINGS TO DO NOW:

Please note that you will be contacted by Wiley Author Services to complete licensing and payment information. The required 'Page Charges Authorization Form' is available here: https://www.embopress.org/pb-assets/embo-site/er_apc.pdf - please download and complete the form and return to embopressproduction@wiley.com

You will receive proofs by e-mail approximately 2-3 weeks after all relevant files have been sent to our Production Office; you should return your corrections within 2 days of receiving the proofs.

Please inform us if there is likely to be any difficulty in reaching you at the above address at that time. Failure to meet our deadlines may result in a delay of publication, or publication without your corrections.

All further communications concerning your paper should quote reference number EMBOR-2021-54540V3 and be addressed to emboreports@wiley.com.

Should you be planning a Press Release on your article, please get in contact with emboreports@wiley.com as early as possible, in order to coordinate publication and release dates.

EMBO Press Author Checklist

Corresponding Author Name: Prof Gavin Hudson and Prof Mary Herbert	
Journal Submitted to: EMBO Reports	
Manuscript Number: EMBOR-2021-54540V1	

USEFUL LINKS FOR COMPLETING THIS FORM
The EMBO Journal - Author Guidelines
EMBO Reports - Author Guidelines
Molecular Systems Biology - Author Guidelines
EMBO Molecular Medicine - Author Guidelines

Reporting Checklist for Life Science Articles (updated January 2022)
This checklist is adapted from Materials Design Analysis Reporting (MDAR) Checklist for Authors. MDAR establishes a minimum set of requirements in transparent reporting in the life sciences (see Statement of Task: 10.31222/osf.io/9sm4x). Please follow the journal's guidelines in preparing your manuscript.
Please note that a copy of this checklist will be published alongside your article.

Abridged guidelines for figures
1. Data
The data shown in figures should satisfy the following conditions:
- the data were obtained and processed according to the field's best practice and are presented to reflect the results of the experiments in an accurate and unbiased manner.
- ideally, figure panels should include only measurements that are directly comparable to each other and obtained with the same assay.
- plots include clearly labeled error bars for independent experiments and sample sizes. Unless justified, error bars should not be shown for technical replicates.
- if n<5, the individual data points from each experiment should be plotted. Any statistical test employed should be justified.
- Source Data should be included to report the data underlying figures according to the guidelines set out in the authorship guidelines on Data Presentation.

2. Captions
Each figure caption should contain the following information, for each panel where they are relevant:
- a specification of the experimental system investigated (eg cell line, species name).
- the assay(s) and method(s) used to carry out the reported observations and measurements.
- an explicit mention of the biological and chemical entity(ies) that are being measured.
- an explicit mention of the biological and chemical entity(ies) that are altered/varied/perturbed in a controlled manner.
- the exact sample size (n) for each experimental group/condition, given as a number, not a range;
- a description of the sample collection allowing the reader to understand whether the samples represent technical or biological replicates (including how many animals, litters, cultures, etc.).
- a statement of how many times the experiment shown was independently replicated in the laboratory.
- definitions of statistical methods and measures:
  - common tests, such as t-test (please specify whether paired vs. unpaired), simple χ2 tests, Wilcoxon and Mann-Whitney tests, can be unambiguously identified by name only, but more complex techniques should be described in the methods section;
  - are tests one-sided or two-sided?
  - are there adjustments for multiple comparisons?
  - exact statistical test results, e.g., P values = x but not P values < x;
  - definition of 'center values' as median or average;
  - definition of error bars as s.d. or s.e.m.

Please complete ALL of the questions below.
Select "Not Applicable" only when the requested information is not relevant for your study.

Materials

Newly Created Materials	Information included in the manuscript?	In which section is the information available? (Reagents and Tools Table, Materials and Methods, Figures, Data Availability Section)
New materials and reagents need to be available; do any restrictions apply?	Not Applicable	

Antibodies	Information included in the manuscript?	In which section is the information available? (Reagents and Tools Table, Materials and Methods, Figures, Data Availability Section)
For **antibodies** provide the following information:<br>- Commercial antibodies: RRID (if possible) or supplier name, catalogue number and or/clone number<br>- Non-commercial: RRID or citation	Not Applicable	

DNA and RNA sequences	Information included in the manuscript?	In which section is the information available? (Reagents and Tools Table, Materials and Methods, Figures, Data Availability Section)
**Short novel DNA or RNA including primers, probes**: provide the sequences.	Not Applicable	

Cell materials	Information included in the manuscript?	In which section is the information available? (Reagents and Tools Table, Materials and Methods, Figures, Data Availability Section)
**Cell lines:** Provide species information, strain. Provide accession number in repository **OR** supplier name, catalog number, clone number, and/**OR** RRID.	Not Applicable	
**Primary cultures:** Provide species, strain, sex of origin, genetic modification status.	Not Applicable	
Report if the cell lines were recently **authenticated** (e.g., by STR profiling) and tested for mycoplasma contamination.	Not Applicable	

Experimental animals	Information included in the manuscript?	In which section is the information available? (Reagents and Tools Table, Materials and Methods, Figures, Data Availability Section)
**Laboratory animals or Model organisms:** Provide species, strain, sex, age, genetic modification status. Provide accession number in repository **OR** supplier name, catalog number, clone number, **OR** RRID.	Not Applicable	
**Animal observed in or captured from the field:** Provide species, sex, and age where possible.	Not Applicable	
Please detail **housing and husbandry conditions**.	Not Applicable	

Plants and microbes	Information included in the manuscript?	In which section is the information available? (Reagents and Tools Table, Materials and Methods, Figures, Data Availability Section)
**Plants:** provide species and strain, ecotype and cultivar where relevant, unique accession number if available, and source (including location for collected wild specimens).	Not Applicable	
**Microbes:** provide species and strain, unique accession number if available, and source.	Not Applicable	

Human research participants	Information included in the manuscript?	In which section is the information available? (Reagents and Tools Table, Materials and Methods, Figures, Data Availability Section)
If collected and within the bounds of privacy constraints report on age, sex and gender or ethnicity for all study participants.	Not Applicable	

Core facilities	Information included in the manuscript?	In which section is the information available? (Reagents and Tools Table, Materials and Methods, Figures, Data Availability Section)
If your work benefited from core facilities, was their service mentioned in the acknowledgments section?	Not Applicable	

Design

Study protocol	Information included in the manuscript?	In which section is the information available? (Reagents and Tools Table, Materials and Methods, Figures, Data Availability Section)
If study protocol has been **pre-registered, provide DOI in the manuscript**. For clinical trials, provide the trial registration number **OR** cite DOI.	Not Applicable	
Report the **clinical trial registration number** (at ClinicalTrials.gov or equivalent), where applicable.	Not Applicable	

Laboratory protocol	Information included in the manuscript?	In which section is the information available? (Reagents and Tools Table, Materials and Methods, Figures, Data Availability Section)
Provide DOI OR other citation details if **external detailed step-by-step protocols** are available.	Not Applicable	

Experimental study design and statistics	Information included in the manuscript?	In which section is the information available? (Reagents and Tools Table, Materials and Methods, Figures, Data Availability Section)
Include a statement about **sample size** estimate even if no statistical methods were used.	Not Applicable	
Were any steps taken to minimize the effects of subjective bias when allocating animals/samples to treatment (e.g. **randomization procedure**)? If yes, have they been described?	Not Applicable	
Include a statement about **blinding** even if no blinding was done.	Not Applicable	
Describe **inclusion/exclusion criteria** if samples or animals were excluded from the analysis. Were the criteria pre-established?<br><br>If sample or data points were omitted from analysis, report if this was due to attrition or intentional exclusion and provide justification.	Not Applicable	
For every figure, are **statistical tests** justified as appropriate? Do the data meet the assumptions of the tests (e.g., normal distribution)? Describe any methods used to assess it. Is there an estimate of variation within each group of data? Is the variance similar between the groups that are being statistically compared?	Not Applicable	

Sample definition and in-laboratory replication	Information included in the manuscript?	In which section is the information available? (Reagents and Tools Table, Materials and Methods, Figures, Data Availability Section)
In the figure legends: state number of times the experiment was **replicated** in laboratory.	Not Applicable	
In the figure legends: define whether data describe **technical or biological replicates**.	Not Applicable	

Ethics

Ethics	Information included in the manuscript?	In which section is the information available? (Reagents and Tools Table, Materials and Methods, Figures, Data Availability Section)
Studies involving **human participants**: State details of **authority granting ethics approval** (IRB or equivalent committee(s), provide reference number for approval.	Not Applicable	
Studies involving **human participants**: Include a statement confirming that **informed consent** was obtained from all subjects and that the experiments conformed to the principles set out in the WMA Declaration of Helsinki and the Department of Health and Human Services Belmont Report.	Not Applicable	
Studies involving **human participants:** For publication of **patient photos**, include a statement confirming that consent to publish was obtained.	Not Applicable	
Studies involving experimental **animals**: State details of **authority granting ethics approva**l (IRB or equivalent committee(s), provide reference number for approval. Include a statement of compliance with ethical regulations.	Not Applicable	
Studies involving **specimen and field samples:** State if relevant **permits** obtained, provide details of authority approving study; if none were required, explain why.	Not Applicable	

Dual Use Research of Concern (DURC)	Information included in the manuscript?	In which section is the information available? (Reagents and Tools Table, Materials and Methods, Figures, Data Availability Section)
Could your study fall under dual use research restrictions? Please check biosecurity documents and list of **select agents and toxins** (CDC): https://www.selectagents.gov/sat/list.htm	Not Applicable	
If you used a select agent, is the security level of the lab appropriate and reported in the manuscript?	Not Applicable	
If a study is subject to dual use research of concern regulations, is the name of the **authority granting approval and reference number** for the regulatory approval provided in the manuscript?	Not Applicable	

Reporting

The MDAR framework recommends adoption of discipline-specific guidelines, established and endorsed through community initiatives. Journals have their own policy about requiring specific guidelines and recommendations to complement MDAR.

Adherence to community standards	Information included in the manuscript?	In which section is the information available? (Reagents and Tools Table, Materials and Methods, Figures, Data Availability Section)
State if relevant guidelines or checklists (e.g., **ICMJE, MIBBI, ARRIVE, PRISMA**) have been followed or provided.	Not Applicable	
For **tumor marker prognostic studies,** we recommend that you follow the **REMARK** reporting guidelines (see link list at top right). See author guidelines, under 'Reporting Guidelines'. Please confirm you have followed these guidelines.	Not Applicable	
For **phase II and III randomized controlled trials**, please refer to the **CONSORT** flow diagram (see link list at top right) and submit the CONSORT checklist (see link list at top right) with your submission. See author guidelines, under 'Reporting Guidelines'. Please confirm you have submitted this list.	Not Applicable	

Data Availability

Data availability	Information included in the manuscript?	In which section is the information available? (Reagents and Tools Table, Materials and Methods, Figures, Data Availability Section)
Have **primary datasets** been deposited according to the journal's guidelines (see 'Data Deposition' section) and the respective accession numbers provided in the Data Availability Section?	Not Applicable	
Were **human clinical and genomic datasets** deposited in a public access-controlled repository in accordance to ethical obligations to the patients and to the applicable consent agreement?	Not Applicable	
Are **computational models** that are central and integral to a study available without restrictions in a machine-readable form? Were the relevant accession numbers or links provided?	Not Applicable	
If publicly available data were reused, provide the respective **data citations in the reference list**.	Not Applicable	
